# RNA N4‐acetylcytidine modification and its role in health and diseases

**DOI:** 10.1002/mco2.70015

**Published:** 2025-01-03

**Authors:** Qiang Wang, Yixiao Yuan, Qiang Zhou, YingDong Jia, Jing Liu, GuangJun Xiao, Chunhong Li, Xiulin Jiang

**Affiliations:** ^1^ Gastrointestinal Surgical Unit Suining Central Hospital Suining Sichuan China; ^2^ Department of Hepatobiliary Surgery The First Affiliated Hospital of Chongqing Medical University Chongqing China; ^3^ Department of Oncology Suining Central Hospital Suining Sichuan China; ^4^ Department of Clinical Laboratory Medicine Suining Central Hospital Suining Sichuan China; ^5^ Department of Medicine UF Health Cancer Center University of Florida Gainesville Florida USA

**Keywords:** human cancer, N4‐acetylcytidine, NAT10, pathology, physiology, RNA modification, tumor immunotherapy

## Abstract

N4‐acetylcytidine (ac4C) modification is a crucial RNA modification widely present in eukaryotic RNA. Previous studies have demonstrated that ac4C plays a pivotal role in viral infections. Despite numerous studies highlighting the strong correlation between ac4C modification and cancer progression, its detailed roles and molecular mechanisms in normal physiological processes and cancer progression remain incompletely understood. This review first outlines the key regulatory enzyme mediating ac4C modification, N‐acetyltransferase 10 (NAT10), including its critical roles in regulating RNA stability, transcriptional efficiency, and translational fidelity. Additionally, it systematically summarizes the essential functions and mechanisms of ac4C modification in normal biological processes, including stem cell fate determination, spermatogenesis and oogenesis, embryonic development, cellular senescence, and bone remodeling. Furthermore, this review delves into the central roles and molecular mechanisms of ac4C modification in regulating malignant proliferation, cell cycle arrest, EMT, drug resistance, cell death, cancer metabolism, and tumor immunotherapy. It also emphasizes the potential of NAT10 as a prognostic biomarker and its therapeutic potential as a target for disease treatment. In summary, this review clarifies the multifaceted roles of ac4C modification in both health and disease and explores NAT10‐targeted therapies with the aim of advancing cancer research and improving patient outcomes.

## Graphical abstract

N4‐acetylcytidine (ac4C) modification is a crucial RNA modification widely present in eukaryotic RNA. This review first outlines the latest research advancements on the roles and mechanisms of ac4C modification in both health and disease and explores ac4C‐targeted therapies with the aim of advancing cancer research and improving patient outcomes

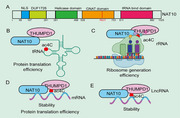

## 1 INTRODUCTION

Epigenetics refers to heritable information transfer between parent and offspring that occurs without alterations in the DNA sequence.^1^, ^2^ It encompasses DNA methylation, histone acetylation, and RNA methylation, among others.^3^ Similarly, numerous chemical modifications exist on RNA, which are ubiquitous in eukaryotes. These modifications were once thought to be static and irreversible. To date, over 100 different types of RNA modifications have been discovered, greatly enhancing the functional diversity and genetic information of RNA.^4^, ^5^, ^6^ Various nucleoside modifications are present in both prokaryotic and eukaryotic RNA. Nucleoside modifications also occur in mRNA, including 7‐methylguanosine, N6‐methyladenosine (m6A), N4‐acetylcytidine (ac4C), methylcytosine, methylguanosine, methyladenosine, and 5‐methylcytosine, among others,^7^, ^8^, ^9^, ^10^ Increasing research suggests that these modifications, as novel posttranscriptional regulatory mechanisms, play critical roles in various physiological and pathological processes. Aberrant regulation of RNA epigenetics is a key factor influencing tumor progression.^4^, ^11^, ^12^ Chemical modifications of mRNA are involved in regulating gene expression, with m6A methylation being the most extensively studied.^13^, ^14^, ^15^, ^16^ Research has shown that m6A modifications are involved in stem cell differentiation,^17^ tissue development,^18^ and tumor progression,^19^, ^20^ underscoring the significance of RNA modifications in tumorigenesis. However, our understanding of other RNA modifications, particularly the role of ac4C modification on mRNA, remains limited.

Initially, ac4C was discovered in the anticodon of bacterial transfer RNA (tRNA).^21^ Studies found that cytidine acetyltransferase (TmcA) catalyzes the formation of ac4C in the presence of acetyl‐CoA and ATP, preventing misreading of the isoleucine codon AUA during protein synthesis and promoting the formation of the tRNA tertiary structure.^22^ This modification was later identified in serine and leucine tRNA, as well as 18S ribosomal RNA (rRNA) in eukaryotes.^23^, ^24^ More recently, ac4C modification was discovered on mRNA. Ac4C plays multiple roles in RNA, such as improving the fidelity of protein translation in tRNA and enhancing the stability and translation efficiency of mRNA.^25^, ^26^ In Saccharomyces cerevisiae, rRNA cytidine acetyltransferase 1 (Rra1p) catalyzes the formation of ac4C1773 using acetyl‐CoA and ATP as substrates.^27^ N‐acetyltransferase 10 (NAT10) is a direct homolog of bacterial TmcA and yeast Rra1p in humans and mice.^28^ It catalyzes the formation of ac4C at position 1842 in the terminal helix of mammalian 18S rRNA in the presence of acetyl‐CoA and ATP, participating in rRNA processing and ribosome formation.^29^ Kre33 is an acetyltransferase that plays a key role in the ribosome biogenesis process in eukaryotes.^30^ It is crucial for the processing and modification of 18S rRNA precursors.^31^ The acetyltransferase activity of Kre33 regulates the structure and function of rRNA through acetylation, thereby affecting protein synthesis.^32^ NAT10, currently the only known acetyltransferase in eukaryotes, has been found to be involved in acetylation modifications of histones, tRNA, 18S rRNA, and mRNA. Structurally, NAT10 contains both an acetyltransferase domain and an RNA‐binding domain, making it the key enzyme responsible for ac4C modification.^10^, ^33^ In 2018, Arango et al.^26^ first discovered that NAT10, by mediating the acetylation of RNA ac4C, maintains mRNA stability and promotes translation rates. Subsequently, an increasing body of literature has demonstrated that NAT10, through RNA ac4C acetylation, participates in various physiological and pathological processes, indirectly influencing disease occurrence and progression.^10^, ^33^, ^34^ However, researchers have yet to fully elucidate and explain the specific pathways and mechanisms by which NAT10 exerts these effects. The role and significance of NAT10 in these diseases and their treatment remain emerging areas in need of further research.

NAT10‐mediated RNA acetylation plays a crucial role in regulating gene expression and participating in various physiological and pathological processes. As scientific research progresses, the study of RNA acetylation has become increasingly prominent. To better understand the different roles of NAT10‐mediated RNA acetylation in normal physiological processes and pathological conditions, we review the research history, detection methods, and biological functions of NAT10‐mediated RNA acetylation. Furthermore, we discuss the roles and mechanisms of NAT10‐mediated RNA acetylation in human health and various diseases, emphasizing the importance of NAT10 as a diagnostic biomarker and a potential therapeutic target. Finally, we summarize the current limitations of RNA acetylation research and propose potential directions for future studies.

This review comprehensive examination of NAT10‐mediated RNA ac4C modification and its roles in health and disease, addressing gaps in understanding the mechanisms and pathophysiological functions of ac4C modification in this field. Although significant progress has been made in RNA modification research, particularly regarding modifications like m6A, the functional roles and regulatory mechanisms of RNA ac4C remain insufficiently elucidated. This review not only summarizes the critical role of NAT10 in ac4C modification but also explores its potential impact on gene expression regulation, cellular functions, and its involvement in various diseases, such as cancer and neurodegenerative disorders. Therefore, this review will lie in synthesizing the latest research advances to provide a comprehensive understanding of RNA ac4C modification and its functional relevance in health and disease, offering new insights and directions for future research and clinical applications.

## 2 ac4C MODIFICATION

Ac4C modification has emerged as a prominent chemical modification in the field of RNA epigenetics in recent years.^31^ Initially, Ac4C modification was primarily discovered in bacterial tRNA and rRNA, and it was later identified in various RNA molecules in eukaryotes.^35^ With further research, Ac4C has been shown to play significant roles in regulating RNA stability and translation efficiency, and it has been closely linked to various physiological and pathological processes.^33^ Particularly, Ac4C modification in mRNA has gained attention for its potential role in gene expression regulation, contributing to tumorigenesis and cancer progression. We will summarize the research history of Ac4C modification and evaluate the advantages and limitations of the methods used for its detection.

### 2.1 The research history of ac4C modification

In 1966, ac4C was first discovered in yeast tRNA.^36^ By 1972, ac4C modification was identified at the wobble position of the methionine elongator tRNA (tRNAMet) in Escherichia coli. It was later demonstrated that ac4C can stabilize the internal C3′ conformation of ribose, facilitating the accurate codon recognition by tRNA. Ac4C was also found at position 12 in yeast leucine tRNA (tRNALeu) and Saccharomyces cerevisiae serine tRNA (tRNASer).^22^ Recent studies have shown that in eukaryotic tRNA, ac4C is restricted to position 12.^37^ In 1978, Thomas et al.^38^ detected ac4C on the small subunit of 18S rRNA in rat, indicating the presence of ac4C in eukaryotic 18S rRNA. By 1993, Bruenger et al.^24^ discovered ac4C modifications in the 5S rRNA of Thermotoga maritima. In human HEK293 cells, NAT10 catalyzes the formation of ac4C at position 1842 of 18S rRNA.^29^ Harada et al.^39^ were the first to report ac4C modification in mammalian cells, detecting ac4C in mature tRNA of HeLa cells using two‐dimensional polyacrylamide gel electrophoresis and sequencing techniques. Sharma et al.^40^ identified two ac4C sites on 18S rRNA in Schizosaccharomyces pombe and human HCT116 cells—one within a helix critical for maintaining translation accuracy and another near an editing site in helix 45. Ikeuchi et al.^41^ further discovered that in the presence of acetyl‐CoA and tRNAMet, TmcA (tRNAMet cytidine acetyltransferase) can stimulate ATP/GTP hydrolysis, catalyzing ac4C formation at the wobble base of bacterial tRNAMet.1. Early ac4C studies largely focused on observing modification sites in tRNA and rRNA. However, in recent years, numerous ac4C modifications have also been identified in mRNA of both humans and yeast.^26^ Another group of researchers used HPLC/MS techniques to detect RNA modifications in the 5S rRNA of thermophiles Sulfolobus and Pyrodictium occultum, revealing ac4C modification in these bacterial 5S rRNAs, which may contribute to their thermal stability.^24^ In 2018, Arango et al.^26^ demonstrated the presence of ac4C in over 4000 regions of the human transcriptome, showing that ac4C is mainly enriched in the coding sequence (CDS) region of human HeLa cells, with its content gradually decreasing from the 5′ to the 3′ end of transcripts. However, as Arango et al.^26^ are the only group to have published findings on ac4C in human mRNA, the reliability of this experiment and its results requires further validation. In 2019, Tardu et al.^42^ found high levels of ac4C in yeast mRNA samples, with ac4C content significantly increasing under oxidative stress conditions.

### 2.2 Detection methods of Ac4C modifications

Advances in techniques for detecting ac4C in RNA molecules have significantly progressed. In earlier times, partial enzymatic hydrolysis and two‐dimensional paper chromatography were used to locate ac4C in rRNA and tRNA.^23^ Recently, combined LC–MS and HPLC–MS analyses have enabled quantitative detection of ac4C in RNA from yeast and human HCT116 cells.^27^ In 2018, Thomas et al.^43^ used sodium borohydride reduction to map ac4C, a method that exploits ac4C's sensitivity to sodium borohydride‐based reduction. Thomas et al.^43^ first extracted total RNA from tissues or cultured cells and treated the RNA with NaBH4 in vitro, inducing mismatched base pairs during reverse transcription (RT). These mismatches cause premature termination during RT, which can be detected and quantified by Sanger sequencing or next‐generation sequencing methods. This method allows for sensitive detection of single ac4C sites from small RNA samples.^43^ However, sodium borohydride reduction cannot analyze ac4C in densely modified RNAs like tRNA. For instance, ac4C sites in eukaryotic tRNA are adjacent to dihydrouridine, and the reduction of ac4C may severely restrict RT read‐through. In 2017, Sinclair et al.^44^ developed an affinity reagent in vitro transcription technique for screening antibodies targeting ac4C‐binding proteins. This method allowed for the artificial synthesis of ac4C‐containing RNA.^44^ Subsequently, Arango et al.^26^ used the acRIP‐seq method, which employs antibodies against ac4C‐binding proteins to enrich ac4C‐modified mRNA, identifying highly enriched ac4C peaks in over 4000 regions and mapping ac4C positions in the human transcriptome for the first time. RNA ac4C modification was first detected in mammalian cells using two‐dimensional polyacrylamide gel electrophoresis and sequencing techniques.^45^ Proton nuclear magnetic resonance spectroscopy has also played a crucial role in analyzing the structural biological features of RNA ac4C modifications.^46^ Recently, dot blot assays^47^ and RedaC: T‐seq have been developed to detect ac4C expression levels in samples.^48^ Despite the numerous detection methods available, researchers must choose the most appropriate approach based on their specific circumstances, as each method has its own strengths and limitations. Currently, no single method can perfectly quantify ac4C levels. Therefore, we systematically summarize the advantages and limitations of different ac4C detection methods. Based on the findings of Arango et al.^26^ and Zhao et al,^49^ an ac4C predictor called PACES was developed to infer ac4C positions in mRNA sequences. However, because the exact mechanism of ac4C formation is still unclear, predicted ac4C sites remain incomplete. Additionally, PACES can only predict the sequence in which ac4C may occur, not its exact location, and since only 4000 human sequences with ac4C have been identified in HeLa cells, the use of PACES to predict ac4C in other species or cell types should be approached with caution. Given that different methods for detecting ac4C modifications each have their own advantages and limitations, we have systematically summarized the currently commonly used techniques for ac4C detection, along with their respective strengths and weaknesses (Table 1).

**TABLE 1:** Summary of advantages and limitations of RNA ac4C detection methods.

Methods	Discovery	Advantages	Limitations	References
HPLC	First observation of abnormal changes in RNA ac4C modification observed in tumor models	Accurate quantitative analysis of ac4C modification	Detects the global level and cannot distinguish specific RNA modification sites; requires large‐scale instruments	^24^
Two‐dimensional polyacrylamide gel electrophoresis and sequencing techniques	First detection of RNA ac4C modification in mammalian cells	RNA ac4C modification at single‐nucleotide resolution	Low throughput; detects only specific RNA modification sites; complex experimental procedures	^39^
Proton nuclear magnetic resonance spectroscopy	The first analysis of structural biology features of RNA ac4C modification	Reveals structural characteristics of nucleic acid modifications	Requires large‐scale instruments and complex operations	^50^
Dot blot assay	Detection of RNA ac4C modification employed ac4C antibody	Simple and fast	Insufficient sensitivity; detects the global level and cannot distinguish specific RNA modification sites	^51^
ac4C‐seq	First high‐throughput detection of RNA ac4C modification in hyperthermophilic archaea at single‐nucleotide resolution	High‐throughput detection of ac4C modification at single‐nucleotide resolution	No detection of ac4C modification in mammalian mRNA, possibly due to insufficient sensitivity; reducing agents may react with other RNA modifications.	^52^
acRIP‐seq	First detection of RNA ac4C modification at a whole‐genome scale in mammalian cells	High‐throughput detection of ac4C modification on cellular transcripts	Requires a large amount of cellular input; unable to precisely locate ac4C modification sites at single‐nucleotide resolution	^53^
RedaC: T‐seq	High‐throughput detection of intracellular RNA ac4C modification at single‐nucleotide resolution	High‐throughput detection of ac4C modification at single‐nucleotide resolution; simple and fas	Sodium borohydride may react with other RNA modifications; requires higher sequencing data volume	^54^

Abbreviations: ac4C‐seq, acetylcytidine sequencing; HPLC, high‐performance liquid chromatography; acRIP‐seq, acetylated RNA immunoprecipitation sequencing; RedaC: T‐seq, reduced representation acetylated cytidine sequencing.

## 3 STRUCTURE AND BIOLOGICAL FUNCTIONS OF NAT10

NAT10 is a multifunctional enzyme that has garnered significant attention in recent years due to its critical role in RNA acetylation. As the only known acetyltransferase in eukaryotes, NAT10 catalyzes the Ac4C modification in tRNA, rRNA, and mRNA. This modification plays a crucial role in regulating RNA stability, translation efficiency, and gene expression.

### 3.1 Structure and localization of NAT10

The gene encoding NAT10 is located on chromosome 11, with a sequence length of 45 kb. The NAT10 protein consists of 1025 amino acids, with a relative molecular weight of approximately 116,000. Its structure includes an acetyltransferase domain, a tRNA‐binding domain, and an RNA helicase domain. The RNA helicase domain is primarily involved in the processing and assembly of 18S rRNA, while mutations in the acetyltransferase domain (R637A) can completely inhibit the acetylation of 18S rRNA and RNA.^22^, ^30^, ^55^ According to the literature, ac4C modification sites mediated by NAT10 occur in the 5′ nuclear localization signal (NLS) sequence of base‐paired regions.^10^ NAT10 is widely expressed in various tissues, including lymphatic tissue, kidney, liver, cerebellum, cerebral cortex, and the central nervous system during embryonic development. In normal tissues, NAT10 is localized in the nucleus; however, in tumor cells, NAT10 shows a translocated expression, being distributed in the cytoplasm, nucleoplasm, and nuclear membrane, suggesting the complexity and broad range of its functions.^33^ Zhang et al.^55^ found that NAT10 is localized in the nucleolus of normal intestinal epithelial cells, whereas in colorectal cancer (CRC) cells, NAT10 translocates to the nucleus, cytoplasm, and cell membrane. Bioinformatics analysis indicates that the subcellular localization of NAT10 is dependent on its NLS sequence (residues 989–1018). Certain mutations in the gene encoding NAT10 (residues 989–1018) can affect its nuclear localization, leading to the accumulation of NAT10 in the cytoplasm. However, some mutations (residues 68–75) do not impact NAT10 localization, and they remain present in both the nucleolus and nucleoplasm. In conclusion, a comprehensive understanding of the structural composition of NAT10 is crucial for elucidating its role in disease progression.

### 3.2 Biological functions of NAT10

**FIGURE 1:**
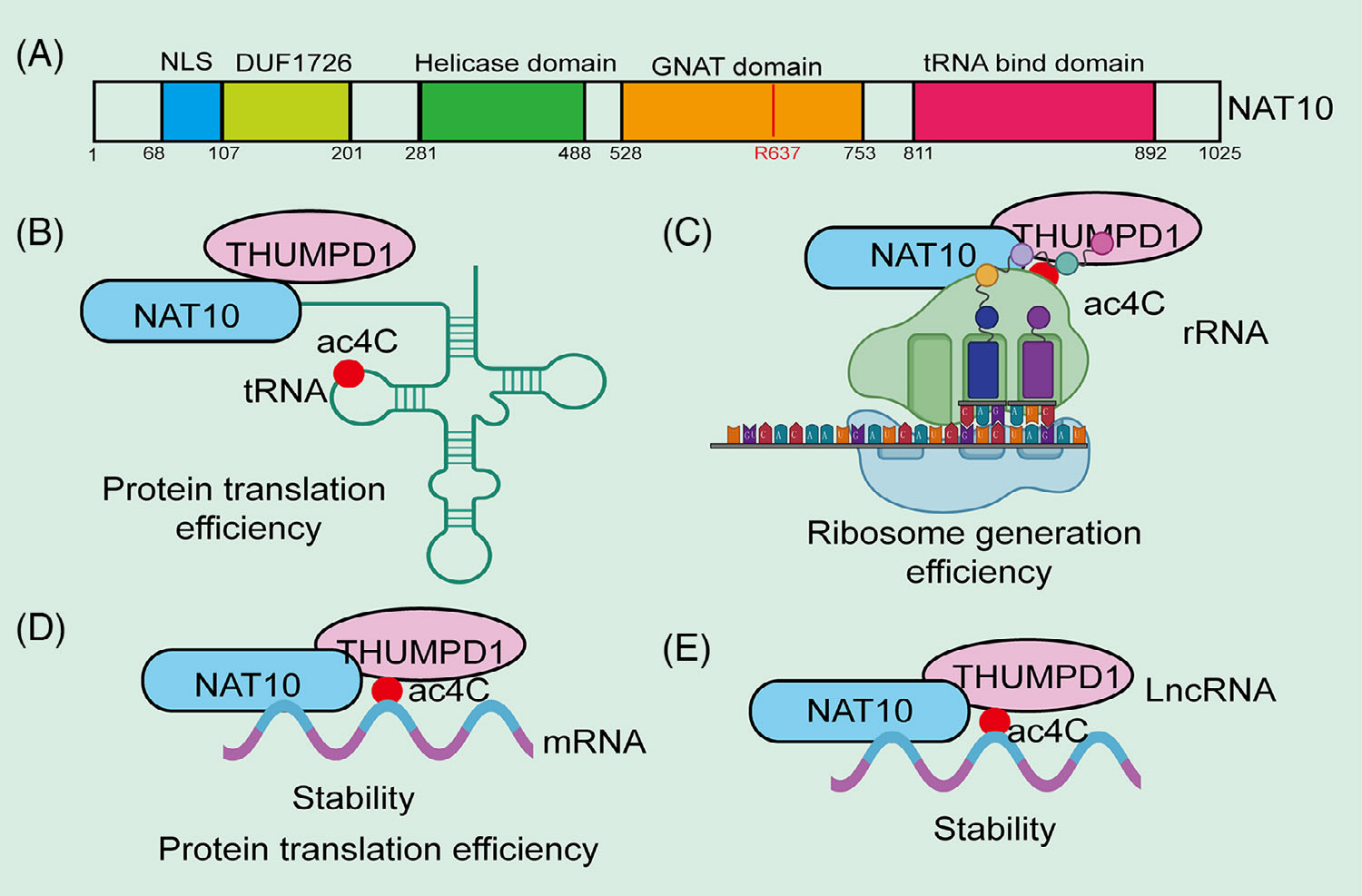
Structural composition and functions of NAT10. (A) Different structural domains of the NAT10 protein and their associated functions. (B) The role of NAT10 in regulating protein translation efficiency. (C) The involvement of NAT10 in the regulation of ribosome biogenesis. (D) The function of NAT10 in modulating protein synthesis efficiency. (E) The role of NAT10 in regulating the stability of noncoding RNAs. NLS, nuclear localization signals.

NAT10 is a member of the GCN5‐related N‐acetyltransferase (GNAT) family of histone acetyltransferases (HATs). Homologous genes of NAT10 in other species include DROME (D. melanogaster), SCHPO (S. pombe), ARATH (A. thaliana), CAEEL (C. elegans), and Kre33 (S. cerevisiae).^56^ In acetylation of tRNA and rRNA, NAT10 requires auxiliary factors such as THUMPD1 and snoRNA, respectively, but no such factors have been identified for ac4C formation in mRNA. Ito et al.^56^ found that Kre33 (a homolog of NAT10) catalyzes the formation of ac4C at position 1773 of 18S rRNA in Saccharomyces cerevisiae. Kre33 has been identified as an ac4C RNA modification enzyme, similar to NAT10, as it catalyzes ac4C formation at position 1842 in yeast 18S rRNA and at position 12 in yeast tRNALeu and tRNASer.^29^, ^30^ Similar to the synergistic interaction between NAT10 and THUMPD1 in humans, Kre33 requires the Tan1 gene to bind tRNA during the acetylation process in Saccharomyces cerevisiae. Both Tan1 and THUMPD1 contain tRNA‐binding domain.^30^ In thermophilic autotrophic bacteria, MTH_RS04295, the homolog of the Tan1 gene, is essential for catalyzing ac4C synthesis on tRNA.^56^ Arango and colleagues^26^ demonstrated that ac4C modifications are present in human HeLa cell mRNA, and the ac4C modification levels in mRNA significantly decrease in NAT10 knockout (KO) cell lines. In yeast, ac4C formation is also linked to the mRNA retention and splicing (RES) complex, which is highly conserved and consists of Bud13p, Snu17p, and Pml1p in yeast. The absence of Bud13p or Snu17p results in significantly reduced ac4C levels in tRNA, while the loss of Pml1p decreases ac4C levels at high temperatures. RES influences the translation of Tan1 by regulating the splicing efficiency of Tan1 precursor mRNA, thereby modulating ac4C formation on tRNA.^57^ Tardu et al.^42^ discovered that under oxidative stress conditions, a large amount of ac4C modification occurs in yeast mRNA. However, in yeast lacking Rra1 (a homolog of NAT10), nearly no ac4C modifications were observed in mRNA under H_2_O_2_‐induced stress. Additionally, the ac4C content in yeast mRNA increases significantly under high oxidative stress, suggesting that ac4C may play a role in the oxidative stress response.^42^ To better understand the biological functions of ac4C modifications, we have summarized recent research progress on the regulation of RNA functions by ac4C modifications (Figure 1).

#### 3.2.1 NAT10 maintains translational fidelity

The accuracy of protein translation is essential for maintaining cellular function and overall organismal health.^58^ During gene expression, the genetic information carried by mRNA is translated into specific amino acid sequences to form functional proteins. Errors in this translation process can lead to the synthesis of abnormal proteins, potentially causing cellular dysfunction, diseases, or even cancer. Such errors can have profound impacts on cellular metabolism, and thus a range of quality control mechanisms exist within cells to ensure the precision and integrity of protein synthesis.^59^ Studies have shown that NAT10‐mediated ac4C modification plays a pivotal role in maintaining the accuracy of protein translation. Studies have shown that ac4C at the wobble position (34th) in the anticodon loop of Escherichia coli tRNAMMet aids in accurately reading noninitiator AUG codons. The ac4C modification at the wobble position reduces the affinity of tRNAMMet for the AUG codon, thereby minimizing codon misreading during protein synthesis.^60^ Additionally, ac4C stabilizes the C3′‐endo conformation of the ribose at the wobble base, promoting the interaction of CG base pairs and ensuring accurate decoding of the AUG codon as methionine. Kumbhar et al.^61^ also found that the distal conformation of the N4‐acetyl side chain of ac4C can prevent the misrecognition of the isoleucine AUA codon during protein translation. In summary, these findings highlight the critical function of ac4C in regulating protein translation fidelity.

#### 3.2.2 NAT10 regulates mRNA stability and translation efficiency

mRNA stability and translation efficiency are key determinants of gene expression levels. mRNA stability affects its lifespan in the cell, dictating how long it can evade degradation and thus how long it can be translated into protein.^62^ More stable mRNAs typically persist longer, supporting multiple rounds of protein synthesis. Both mRNA stability and translation efficiency together determine the rate and quantity of protein synthesis, which is crucial for cellular function and the normal operation of organisms.^26^ Research has shown that NAT10‐mediated ac4C modification is vital for maintaining mRNA stability and translation efficiency. Posttranscriptional regulation, such as mRNA stability and translation efficiency, is increasingly recognized as a crucial mechanism in gene expression.^63^ In 2018, Arango and colleagues^26^ discovered that ac4C is widely present in the human transcriptome and that NAT10 is the key enzyme catalyzing ac4C modifications on mRNA. Further studies revealed that NAT10 catalyzes ac4C modifications in the CDS region of mRNA, and inhibition of NAT10 reduces ac4C modification levels, leading to decreased mRNA stability and translation efficiency, which is related to its effect on codon preference.^26^ Dominissini et al.^64^ found that NAT10‐mediated ac4C modification can also influence codon–anticodon interactions, thereby regulating mRNA stability and translation efficiency.^64^ In conclusion, these findings suggest that ac4C plays a significant role in regulating mRNA stability and translation efficiency.

#### 3.2.3 NAT10 regulates tRNA stability and cellular thermotolerance

The stability of tRNA is closely linked to cellular thermotolerance. Under heat or other stress conditions, cells must maintain their protein synthesis machinery to ensure survival.^65^ As a key molecule in protein synthesis, tRNA is responsible for transporting amino acids to ribosomes and facilitating peptide chain assembly. tRNA stability determines whether it can function effectively under extreme conditions.^66^ If tRNA degrades or undergoes structural changes at high temperatures, protein synthesis will be disrupted, ultimately impairing cell viability. These mechanisms work together to maintain tRNA functionality in harsh environments, thereby enhancing cellular thermotolerance and helping cells cope with environmental stress. Studies have shown that NAT10‐mediated ac4C modification is critical for regulating tRNA stability and cellular thermotolerance. Johansson and Byström^67^ found that ac4C levels and tRNASerCGA abundance were reduced in Saccharomyces cerevisiae mutants lacking the Tan1 gene, suggesting that ac4C and Tan1 play a critical role in maintaining the stability of mature tRNASerCGA. Xu et al.^68^ demonstrated that the inactivation of catalytic sites on the yeast Tan1 gene led to decreased ac4C levels and reduced tRNASerCGA abundance. Bruenger and colleagues^24^ found that ac4C and ac4Cm were present in the same sequences of 5S rRNA from two thermophilic bacterial strains, suggesting a potential link between ac4C and thermotolerance.^68^ Kawai et al.^50^ analyzed the structural features of ac4Cm in extreme thermophilic tRNA and found that the 2′‐O‐methylation of cytidine (e.g., ac4C) stabilized the C3′‐endo conformation of tRNA, contributing to the thermotolerance of extreme thermophilic tRNA. In 2019, Orita et al.^69^ randomly inserted mutations into thermophilic archaea via artificial transposons and discovered that the absence of certain nucleoside modifications in tRNA resulted in mutants with reduced thermotolerance. However, they did not observe a significant decrease in the melting point of tRNA lacking ac4C. Collectively, this evidence indicates that ac4C plays a crucial role in maintaining tRNA stability and is associated with high cellular thermotolerance.^69^

#### 3.2.4 NAT10 regulates rRNA synthesis

rRNA synthesis is a key step in ribosome biogenesis and cellular protein synthesis.^70^ rRNA is transcribed by RNA polymerase I in the nucleolus and forms the structural and functional core of the ribosome. The amount of rRNA synthesized directly influences the number of ribosomes, thereby regulating the cell's translation capacity and protein synthesis levels.^71^ rRNA synthesis is modulated by multiple cellular signaling pathways, including those related to nutrient availability, cell growth signals, and stress responses. When growth factors and nutrients are abundant, rRNA synthesis accelerates to support rapid cell growth and division. Conversely, under stress conditions or nutrient deprivation, rRNA synthesis slows down to conserve energy and resources.^72^ Additionally, the regulation of rRNA synthesis involves epigenetic modifications, such as chromatin state and DNA methylation, ensuring that rRNA production aligns with cellular needs. Through precise regulation of rRNA synthesis, cells can balance protein synthesis demands with energy consumption, maintaining normal physiological function and adapting to environmental changes. In the nucleolus of eukaryotic cells, pre‐rRNA undergoes synthesis and processing to form various components of the ribosome. The synthesis of pre‐rRNA is regulated by transcriptional U three proteins (t‐UTPs).^73^ NAT10 is one of the t‐UTPs and is localized around chromosomes during mitosis, playing a key role in 18S rRNA synthesis.^74^ Kong et al.^75^ found that NAT10 is localized in the nucleolus and is essential for 18S rRNA processing. Knockdown of NAT10 expression reduces 47S pre‐rRNA levels.^75^ Other studies have shown that NAT10 acetylates 18S rRNA, thereby regulating its synthesis.^27^ Ito et al.^29^ confirmed that RNAi‐mediated knockdown of NAT10 leads to slow cell growth and accumulation of 18S rRNA precursors, which is associated with NAT10‐catalyzed formation of ac(4)C1842 in 18S rRNA. Cai et al.^76^ discovered that K426 is an acetylation site of NAT10 and is essential for activating rRNA transcription. Mutation of K426 in NAT10 disrupts acetylation of upstream binding factors, impairing the interaction between RNA polymerase I‐associated factor 53 and RNA polymerase I, thereby inhibiting pre‐rRNA transcription.^76^ In summary, these findings collectively indicate that ac4C plays an essential role in regulating rRNA synthesis.

#### 3.2.5 NAT10 regulates lncRNA functions

NAT10 can enhance mRNA stability or translation efficiency, but the role of ac4C modifications in the regulation of other noncoding RNAs in mammals remains largely unexplored. Notably, a recent study revealed for the first time the presence of ac4C acetylation modifications in lncRNA. Researchers found that NAT10 upregulates ac4C acetylation on lncRNA CTC‐490G23.2, thereby enhancing the stability of CTC‐490G23.2 RNA. This promotes the binding of CD44 pre‐mRNA to polypyrimidine tract‐binding protein 1 (PTBP1), leading to an increase in CD44v(8‐10) isoforms and promoting tumor invasion and metastasis.^77^ This study provides valuable insight into ac4C acetylation as a regulatory mechanism for lncRNA expression and a potential prognostic biomarker. Conversely, NAT10 is also regulated by lncRNA. For instance, LINC00623 is significantly upregulated in pancreatic ductal adenocarcinoma (PDAC), promoting tumorigenicity and migration of PDAC cells. LINC00623 directly binds to NAT10, recruiting the deubiquitinase USP39 to inhibit the ubiquitin‐mediated degradation of NAT10, thereby maintaining NAT10 protein stability and upregulating downstream mRNA ac4C modifications, ultimately promoting PDAC proliferation, tumorigenesis, migration, and invasion.^78^ Whether ac4C modifications exist in circRNA and miRNA remains unknown and requires further exploration. In conclusion, this evidence suggests that ac4C plays a crucial role in regulating noncoding RNA stability.

#### 3.2.6 Interaction between ac4C and other RNA modifications

ac4C modification not only plays significant roles on its own but also interacts with other RNA modifications to coregulate important biological processes. Interestingly, recent studies have highlighted a notable interaction between ac4C and m6A modifications. For instance, in human osteosarcoma tissues, NAT10 knockdown significantly upregulates m6A modification levels while inhibiting the growth, migration, and invasion of osteosarcoma cells. Further investigations revealed that NAT10 knockdown significantly reduces the mRNA stability and translation of YTHDC1, a reader of m6A‐modified mRNAs. YTHDC1 recognizes m6A modifications in a manner dependent on m6A in key glycolytic enzymes such as phosphofructokinase (PFKM) and lactate dehydrogenase A (LDHA), enhancing the stability of their mRNAs and thus inhibiting the glycolytic pathway. This study was the first to elucidate the interaction between ac4C and m6A modifications and their role in the regulation of glucose metabolism.^79^ Moreover, a recent study revealed previously uncharacterized biological functions of NAT10, demonstrating that NAT10 regulates m6A‐modified target genes via liquid–liquid phase separation (LLPS), thereby promoting gastric cancer progression. Specifically, NAT10 was found to be significantly upregulated in gastric cancer, enhancing cell proliferation, migration, invasion, and the growth of patient‐derived organoids, ultimately accelerating tumor progression. Mechanistically, the study demonstrated that the C‐terminal intrinsically disordered region of NAT10 mediates LLPS and interacts with the splicing factor SRSF2, promoting its acetylation and increasing its stability. Acetylated SRSF2 directly binds to the pre‐mRNA of YTHDF1, regulating its alternative splicing and upregulating its expression, thereby promoting the malignant progression of gastric cancer.^80^ These studies collectively demonstrate that the crosstalk between ac4C and other RNA modifications plays an important role in regulating RNA metabolism and tumor progression.

## 4 NAT10 ROLE IN NORMAL PHYSIOLOGICAL PROCESSES

**FIGURE 2:**
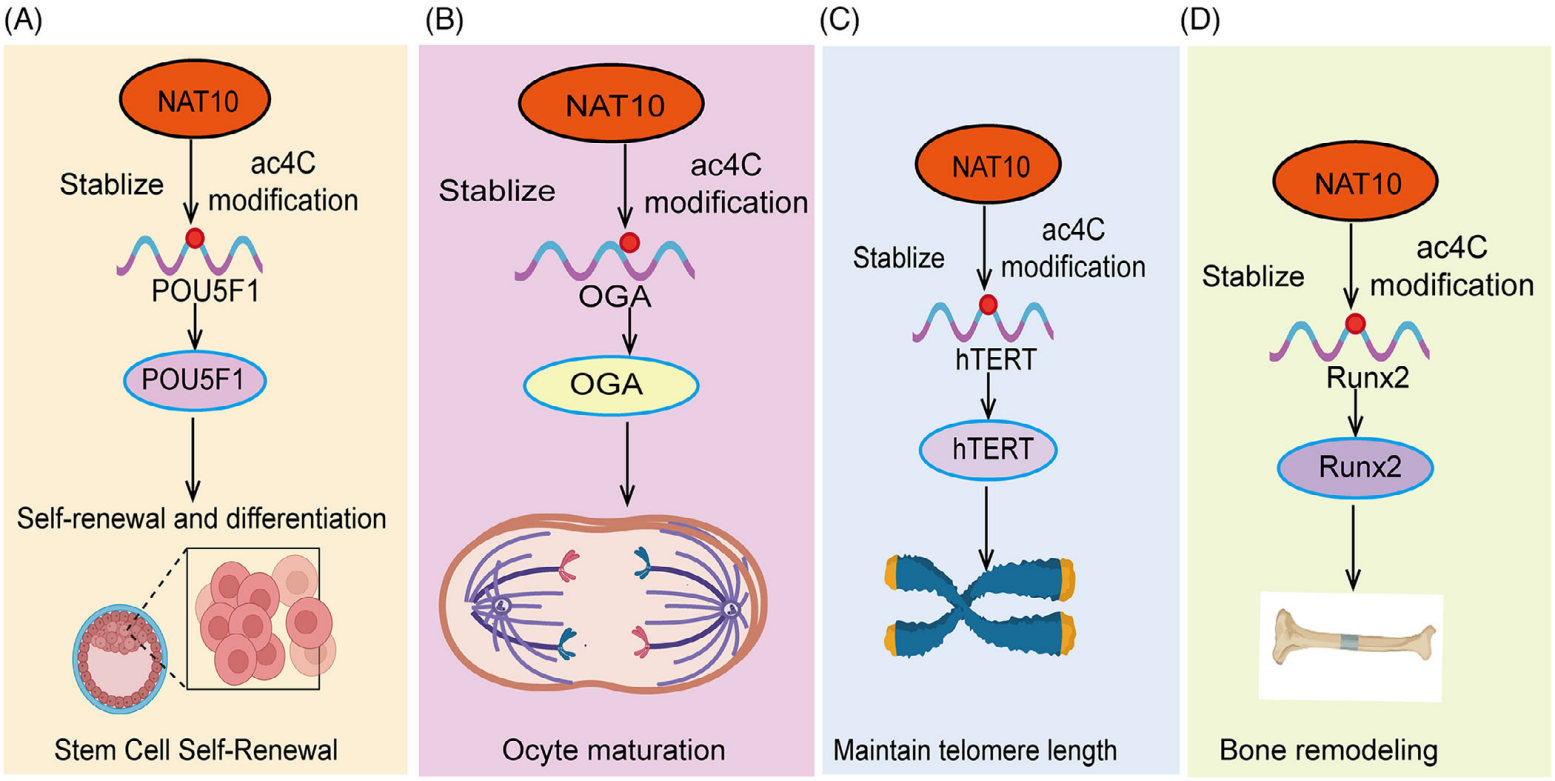
Functions of NAT10‐mediated AC4C modification in normal physiological processes. (A) The involvement of NAT10‐mediated AC4C modification in stem cell fate determination. (B) The role of NAT10‐mediated AC4C modification in embryonic development. (C) The participation of NAT10‐mediated AC4C modification in telomere stability maintenance. (D) The contribution of NAT10‐mediated AC4C modification to bone remodeling. hTERT, human telomerase reverse transcriptase; OGA, O‐GlcNAcase.

NAT10 plays multiple regulatory roles in normal physiological processes, primarily through its acetyltransferase activity, which facilitates acetylation of RNA and proteins. NAT10‐mediated Ac4C modification is involved in the regulation of various biological processes, including stem cell fate, spermatogenesis and oogenesis, embryonic development, cell aging, DNA damage repair, cell cycle progression, chromosome decondensation, autophagy, and bone remodeling (Figure 2). By modulating these essential biological pathways, NAT10 ensures the maintenance of normal cellular function and the overall health of the organism.

### 4.1 NAT10 regulates stem cell fate

Stem cell fate determines the specific type of cell a stem cell will differentiate into during particular developmental stages, ultimately forming various tissues and organs.^81^ Stem cells possess the abilities of self‐renewal and multilineage differentiation, which are fundamental for maintaining tissue homeostasis and repairing damage.^82^ The determination of stem cell fate is regulated by multiple internal and external factors, including genetic information, intracellular signaling pathways, microenvironment, and external stimuli. Epigenetic modifications play a crucial role in regulating stem cell fate. NAT10 is a ribosome‐associated acetyltransferase that plays a critical role in various biological processes, including RNA acetylation, cell cycle regulation, and gene expression control.^51^, ^83^, ^84^ Notably, in the context of stem cell fate determination, NAT10 regulates mRNA stability and translation efficiency through ac4C modification, thereby influencing the expression of specific genes and directing the differentiation pathways of stem cells.^85^ For example, recent studies have revealed the crucial role of NAT10‐catalyzed ac4C epitranscriptional modification of messenger RNA in regulating stem cell fate. In this study, researchers utilized a pluripotent stem cell system and conducted experiments on somatic cell reprogramming and stem cell differentiation into pancreatic lineages to systematically investigate the role of NAT10 in controlling stem cell fate^86^ (Figure 2A). Through ac4C‐RIP and proteomic screening, the researchers identified that NAT10‐mediated ac4C modifications are enriched in chromatin regulators that play a pivotal role in guiding cell fate and identified ANP32B as a downstream functional target. NAT10 regulates the expression level of ANP32B in an ac4C‐dependent manner, and phenotypic experiments further revealed the critical role of the NAT10–ac4C–ANP32B axis in the regulation of cell fate transitions. Integrative multiomics analysis showed that ANP32B partially mediates NAT10 regulation of histone bivalent modifications and chromatin accessibility, thereby precisely controlling stem cell fate.^86^ A similar study demonstrated that NAT10‐mediated ac4C modification enhances the translation efficiency of NR2F1 mRNA, thereby maintaining the ectodermal differentiation of human embryonic stem cells (hESCs). This study reveals a novel regulatory role of ac4C modification in the early ectodermal differentiation of hESCs and offers new strategies for the treatment of neuroectodermal deficiency disorders.^87^ Moreover, NAT10 may affect the self‐renewal and differentiation potential of other types of stem cells through the modulation of ac4C modifications. In summary, the pivotal role of NAT10 in stem cell fate determination positions it as a key target for studying the mechanisms of stem cell differentiation and for developing therapeutic strategies for related diseases.

### 4.2 NAT10 regulation of spermatogenesis and oogenesis

Spermatogenesis and oogenesis are foundational to reproduction, involving the combination of sperm and eggs to transfer genetic material and initiate new life. The proper execution of these processes is critical for the reproductive health and genetic stability of offspring.^88^ Research has shown that RNA modifications are essential for maintaining spermatogenesis and oogenesis. The acetyltransferase enzyme NAT10, which catalyzes ac4C modifications, is highly expressed in spermatogonia, spermatocytes during the prophase of the first meiotic division, and supporting cells, playing key roles in chromosome synapsis, recombination, and segregation.^89^ In male mice, NAT10 deficiency leads to transcriptional dysregulation and downregulation of key genes involved in meiosis, causing meiotic arrest and failure to produce mature sperm, ultimately resulting in infertility.^89^ This underscores the critical importance of ac4C RNA acetylation in maintaining normal spermatogenesis. During oocyte maturation, NAT10‐mediated ac4C acetylation also plays an essential role.^90^ Research shows that from the germinal vesicle stage to the metaphase II (MII) stage, the levels of NAT10 and ac4C modification decrease. When NAT10 is absent, adenylate cyclase 3 is dysregulated during oocyte maturation, leading to a delay in meiosis and a reduced first polar body extrusion rate.^90^ Another study identified O‐GlcNAcase (OGA) as a key target gene for ac4C modification, mediated by NAT10. OGA interacts with G‐protein‐coupled receptors, molecular transduction, and nucleosome DNA binding, and its absence inhibits oocyte maturation^91^ (Figure 2B). These findings highlight the importance of ac4C acetylation in oogenesis, though further investigation is required to uncover more regulatory mechanisms. Recent research uncovered the role of NAT10 in mouse oocyte development, where a Nat10 KO model showed that oocytes arrested at the metaphase I (MI) stage and follicular development halted at the secondary follicle stage. Mechanistic studies revealed that NAT10 regulates the expression of key genes in the CCR4–NOT complex, shortening mRNA poly(A) tails to degrade maternal mRNA, remodeling the maternal transcriptome, and supporting oocyte meiotic growth and maturation.^92^ In summary, these evidences indicate that ac4C plays a critical role in regulating spermatogenesis and oogenesis.

### 4.3 NAT10 regulation of embryonic development

The normal progression of embryonic development is crucial for the health of the organism and reproductive success.^93^ This process is regulated by both genetic and environmental factors, including gene expression, cellular signaling, and nutritional support.^94^ Studying the mechanisms of embryonic development is important for understanding human health and disease. ESCs, which possess self‐renewal and pluripotency, show a decreasing trend in NAT10 expression as the three embryonic germ layers develop and differentiate. NAT10 knockdown in human ESCs leads to an inability to maintain typical undifferentiated colony morphology, with a marked inhibition of cell proliferation and an upregulation of markers related to germ layer differentiation. Further research demonstrated that NAT10 upregulates ac4C modifications on OCT4 mRNA, stabilizing its transcript and enhancing protein expression, thus influencing the self‐renewal and differentiation of hESCs.^85^ In summary, these evidence indicate that ac4C plays a key role in regulating embryonic development.

### 4.4 NAT10 regulation of aging

Human telomerase is a complex composed of RNA and protein, primarily including three subunits: human telomerase reverse transcriptase (hTERT), RNA components, and associated proteins. In 2003, Lv et al.^95^ first discovered that NAT10 binds to the hTERT gene promoter region (–201 to –56 nt), activating hTERT transcription. Further research confirmed that NAT10 overexpression affects telomerase assembly and localization, enhancing its catalytic activity^95^ (Figure 2C). Additionally, the specific small molecule inhibitor Remodelin has been shown to effectively restore nuclear shape in laminopathies by reorganizing microtubules, offering new insights into the function of NAT10 in nuclear structure regulation and its potential as an antiaging therapeutic target.^96^ Further studies found that reducing NAT10 expression inhibits RUNX2 acetylation, thereby promoting mRNA degradation and reducing protein expression, thus inhibiting osteoporosis^97^ (Figure 2D). In studies on aging and Hutchinson‐Gilford progeria syndrome (HGPS), NAT10 KO in mice resulted in embryonic lethality at E14.5, while heterozygous mice (NAT10±) appeared relatively healthy. Notably, NAT10 deficiency led to changes in gene expression in heart tissue, with differentially expressed genes enriched in aging and lifespan‐related pathways. Mechanistic studies suggested that NAT10 deficiency directly downregulates K40 acetylation of α‐tubulin, inhibiting HGPS progression.^98^ NAT10 also suppresses the HGPS phenotype by regulating the nuclear‐cytoplasmic distribution of TNPO1 (transportin‐1), maintaining nuclear pore complex integrity, and Ran GTP subcellular localization.^99^ In summary, these evidence indicate that ac4C plays a crucial role in regulating cell aging.

### 4.5 NAT10 regulation of DNA damage repair

DNA damage repair is an essential mechanism for maintaining genomic stability and cellular function.^100^ Throughout the cell lifecycle, DNA frequently suffers damage from internal and external sources, such as ultraviolet radiation, chemical agents, and oxidative stress. DNA damage repair mechanisms operate at various stages of the cell cycle and across different cell types to ensure DNA integrity and stability.^101^ Cells regulate these repair mechanisms to respond to environmental challenges, thereby preventing disease and ensuring accurate transmission of genetic information. In response to DNA damage caused by various physical and chemical factors, cells activate damage response pathways. NAT10 has been shown to play a role in repairing DNA damage and preventing apoptosis. In studies by Ling et al.,^102^ H_2_O_2_ was used to induce DNA damage in HeLa cells, and RT‐PCR analysis revealed that NAT10 expression increases following DNA damage, enhancing the cells’ resistance to H_2_O_2_‐induced senescence. Additionally, Liu et al.^103^ discovered that NAT10 is a novel regulator of p53 activation. Following DNA damage, NAT10 translocates to the nucleus, where it acetylates p53 at K120, promoting Mdm2 degradation and counteracting Mdm2‐mediated inhibition of p53, thereby preventing apoptosis.^103^ NAT10 expression increases in a time‐ and dose‐dependent manner following DNA damage induced by H_2_O_2_ or cisplatin, with high NAT10 expression enhancing DNA repair and apoptosis resistance.^104^ In summary, these evidence indicate that ac4C plays a critical role in regulating DNA damage repair.

### 4.6 NAT10 regulation of cell cycle progression

The midbody, a transient structure formed during late mitosis in mammals, plays a key role in cell division and is supported by microtubules.^105^ During late mitosis, the nucleolus degrades, and NAT10 relocates from the nucleolus to the midbody. Shen et al.^106^ found that inhibiting NAT10 expression results in nucleolar assembly defects, reduced α‐tubulin acetylation, and disrupted cytokinesis, leading to G2/M phase cell cycle arrest. This suggests that NAT10 may regulate cell division by promoting midbody reorganization during late mitosis and stabilizing α‐tubulin.^106^ In summary, these evidence indicate that ac4C plays a key role in regulating cell cycle progression.

### 4.7 NAT10 regulation of chromosome decondensation

Chromosome decondensation is not only a marker of normal cell cycle progression but also a crucial step for cells to restore normal functions and gene expression after re‐entering interphase.^107^ Abnormalities in this process can lead to disrupted gene expression, chromosomal instability, and impaired cellular function and proliferation. Therefore, precise regulation of chromosome decondensation is vital for maintaining cellular health and genetic stability. After cells enter mitosis, the nucleolus degrades, and NAT10 relocates to the chromosomal periphery. During late mitosis in mammals, DNA is highly condensed to facilitate the separation of sister chromatids, but chromosome decondensation is necessary during telophase to resume transcription for the next cell cycle. Although factors involved in this process remain unclear, acetylation of lysine residues on histones H2B and H4 is associated with chromosome decondensation. In 2007, Chi et al.^108^ demonstrated that chromosome decondensation requires a membrane‐associated HAT, specifically NAT10. Disrupting NAT10 function prolongs chromosome decondensation, while overexpression accelerates it. NAT10 also requires cooperation with the inner nuclear membrane protein hsSUN1, which interacts with separated sister chromatids during late mitosis. Knockdown of hsSUN1 reduces histone acetylation and delays chromosome decondensation, potentially due to hsSUN1 facilitating NAT10 recruitment to chromosomes.^108^ In summary, these evidence indicate that ac4C plays a key role in regulating chromosome decondensation.

### 4.8 NAT10 regulation of autophagy

Autophagy is an important cellular degradation and clearance mechanism that allows cells to handle damaged organelles, protein aggregates, and other intracellular waste.^109^ It is crucial for maintaining cellular homeostasis and plays a key role in cell growth, development, and stress responses.^110^ NAT10 plays a role in the transition between rRNA biogenesis and autophagy. NAT10 acetylation activates rRNA biogenesis and inhibits autophagy induction. Under nutrient‐rich conditions, NAT10 binds and acetylates autophagy regulator Che‐1 at K228, inhibiting Che‐1‐mediated transcriptional activation of downstream genes Redd1 and Deptor, which are inhibitors of mTOR signaling. mTOR promotes cell growth by activating anabolic processes such as rRNA biogenesis and protein synthesis, thereby inhibiting autophagy. Che‐1 is a key autophagy regulator as it inhibits mTOR activity by upregulating Deptor and Redd1 expression. Under nutrient deprivation, such as glucose deficiency, NAT10 becomes a substrate for Sirt1, which deacetylates NAT10 and promotes Che‐1‐mediated transcriptional activation of Redd1 and Deptor, thus inducing autophagy to provide essential nutrients for cell survival.^111^ In summary, these pieces of evidence indicate that ac4C plays a critical role in regulating autophagy.

### 4.9 NAT10 in bone remodeling regulation

Bone remodeling is a dynamic process within the skeletal system that involves continuous remodeling and renewal of bone tissue to maintain bone health and function.^112^ This process involves two main cell types: osteoclasts (bone resorption) and osteoblasts (bone formation). Understanding the mechanisms and regulation of bone remodeling is important for the prevention and treatment of bone diseases. Recent research has shown that NAT10 expression and overall RNA ac4C levels are significantly downregulated in bone tissues of osteoporosis patients. Increasing NAT10 expression can alleviate bone loss while inhibiting NAT10 expression promotes bone mass loss in mice. In addition, elevating ac4C modification levels in bone marrow mesenchymal stem cells can promote calcium nodule formation. In summary, these pieces of evidence indicate that ac4C plays a key role in regulating bone remodeling.

## 5 FUNCTIONS AND MECHANISMS OF NAT10 IN CANCER

**FIGURE 3:**
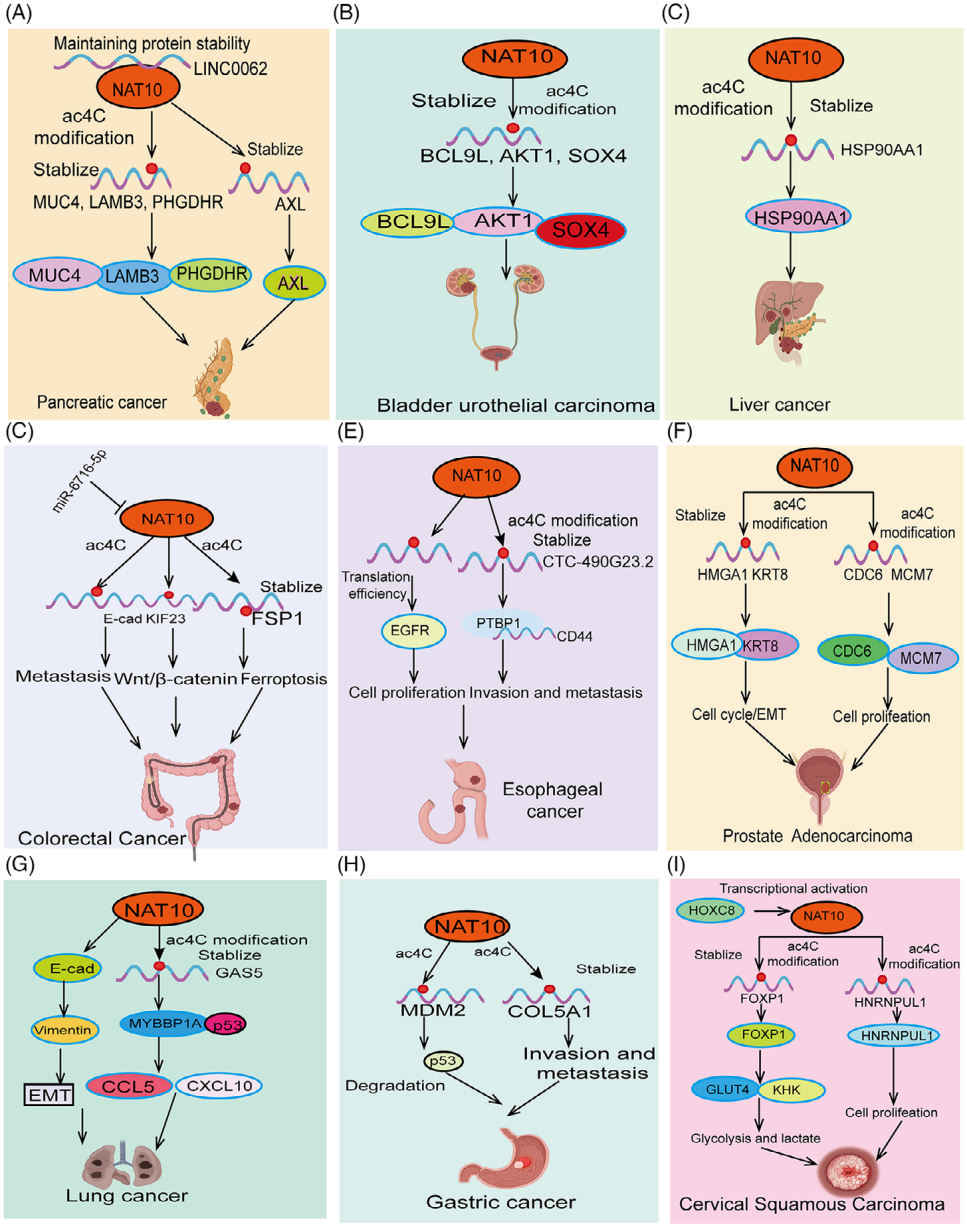
Functions and mechanisms of NAT10‐mediated AC4C modification in various cancers. The roles and mechanisms of NAT10‐mediated AC4C modification in (A) pancreatic cancer, (B) renal cancer, (C) liver cancer, (D) colorectal cancer, (E) esophageal cancer, (F) prostate cancer, (G) lung cancer, (H) gastric cancer, and (I) cervical cancer. AKT, protein kinase B (PKB), often referred to as AKT; CDC6, cell division cycle 6; EMT, epithelial–mesenchymal transition; MUC4, Mucin 4.

**FIGURE 4:**
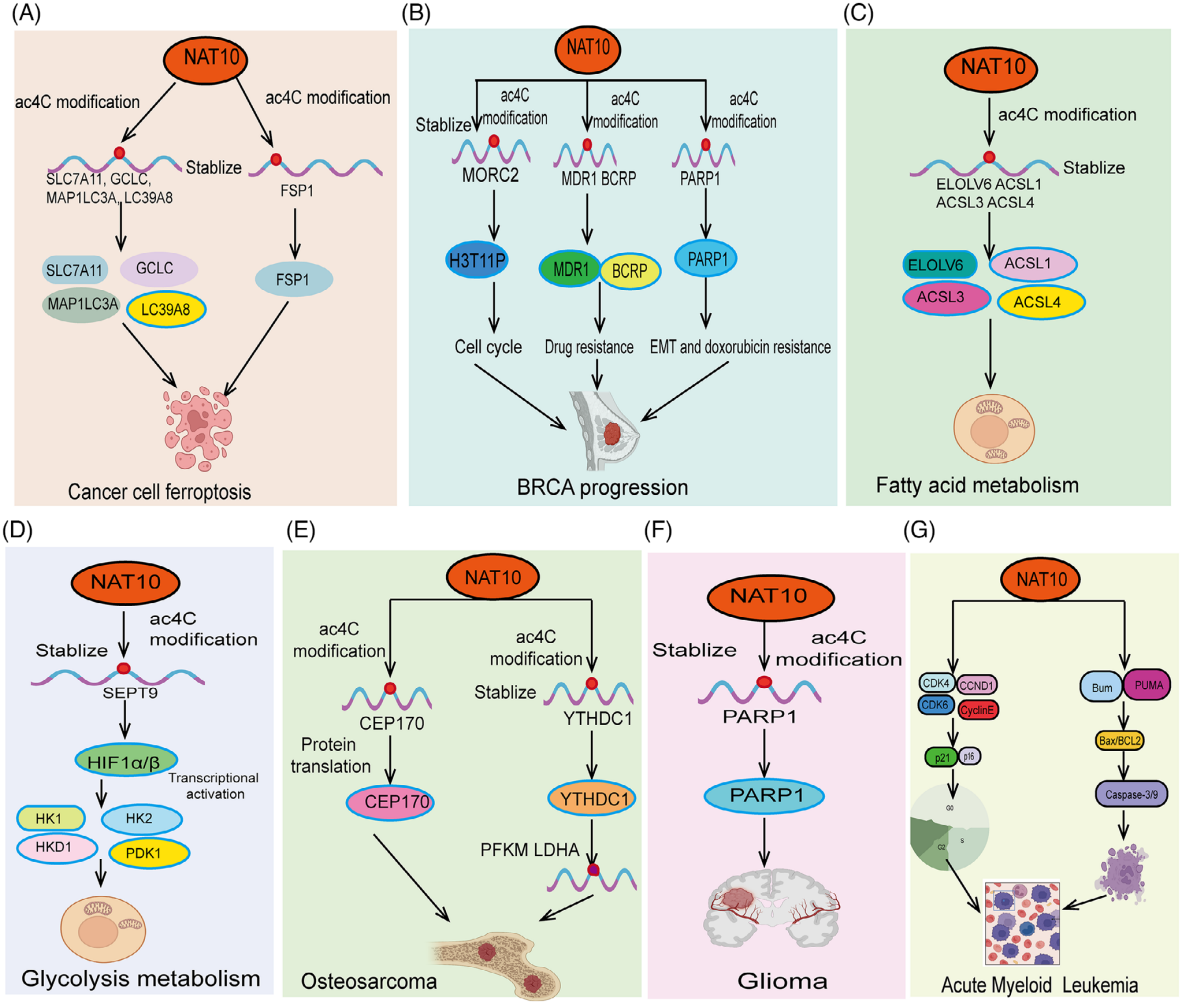
Functions and mechanisms of NAT10‐mediated AC4C modification in cancer cell metabolism and different cancers. The roles and mechanisms of NAT10‐mediated AC4C modification in (A) cancer cell metabolism, (B) breast cancer, (C) fatty acid metabolism, (D) glucose metabolism, (E) osteosarcoma, (F) glioma, and (G) acute myeloid leukemia (AML). GCLC, glutamate‐cysteine ligase catalytic subunit; FSP1, ferroptosis suppressor protein 1; P21, cyclin‐dependent kinase inhibitor 1A (CDKN1A); P16, cyclin‐dependent kinase inhibitor 2A (CDKN2A); HK1, hexokinase 1; HK2, hexokinase 2.

NAT10 mediates ac4C acetylation, playing a crucial role in tumor progression. As a major ac4C‐modifying enzyme, NAT10 regulates the expression of multiple genes related to tumor progression through acetylation. Studies have shown that NAT10‐mediated ac4C acetylation is highly expressed in various cancers, promoting cancer cell proliferation, invasion, and metastasis by regulating metabolic pathways, enhancing stress response, and affecting genes related to the cell cycle and apoptosis (Figures 3 and 4). Moreover, We summarized the outstanding functions and molecular mechanisms of NAT10 in regulating ferroptosis, fatty acid metabolism, glucose metabolism, and tumor immunity.

### 5.1 NAT10 regulation of tumor cell proliferation

The unlimited proliferation of tumor cells is a hallmark of cancer progression.^113^ Tumor cells gain the ability to proliferate continuously through abnormal regulation of the cell cycle, enabling rapid expansion and accumulation of cancer cells.^114^ These processes collectively drive cancer progression and exacerbation, making tumors difficult to control and closely related to patient prognosis. Recent studies have shown that abnormal acetylation of ac4C is often associated with malignant proliferation of tumor cells. Studies have shown that NAT10 is significantly upregulated in esophageal cancer (ESCA). Patients with high NAT10 expression have significantly shorter survival times. Inhibiting NAT10 expression in ESCA cell lines significantly suppresses tumor progression. Mechanistic studies suggest that NAT10 regulates tRNA abundance in an ac4C RNA‐dependent manner, promoting EGFR protein translation efficiency and facilitating ESCA tumor malignancy.^28^ Studies have shown that LINC0062 is upregulated in PDAC tissues. Overexpression of LINC0062 promotes tumor migration and growth. Further analysis found that LINC0062 directly binds to NAT10, protecting it from ubiquitination degradation and maintaining its protein stability, thereby regulating the expression of downstream target genes MUC4, LAMB3, and PHGDHR in an acetylation modification‐dependent manner, promoting PDAC malignancy^78^ (Figure 3A). High NAT10 expression is observed in bladder urothelial carcinoma (BLCA) and knocking down NAT10 expression significantly inhibits BLCA tumor cell growth and promotes apoptosis. Further research confirmed that NAT10 functions as a classical acetyltransferase, directly binding to the transcripts of BCL9L, cell cycle (AKT1), and stem cell maintenance (SOX4), increasing their acetylation levels, thereby enhancing their stability and translation efficiency^33^ (Figure 3B). Consistently, another study showed that NAT10 mRNA and protein levels are highly upregulated in PDAC tissues and are associated with shorter survival times in PDAC patients. In vivo and in vitro experiments demonstrated that NAT10 promotes acetylation modification of AXL mRNA, increasing its transcript stability, and promoting PDAC malignant proliferation, and distant metastasis.^115^ Additionally, a recent study revealed that high NAT10 expression is associated with increased liver cancer risk and poor prognosis. Elevated NAT10 expression significantly enhances the metastatic ability and antiapoptotic capacity of HCC cells under endoplasmic reticulum stress (ERS). Mechanistic studies found that NAT10 directly binds to HSP90AA1, increasing the ac4C modification level of HSP90AA1 mRNA, thereby maintaining its stability and upregulating its expression (Figure 3C). Another study demonstrated significant upregulation of NAT10 in LSCC. Knockdown of NAT10 markedly suppressed proliferation, migration, and invasion of LSCC cells. Acting as its downstream target gene, NAT10 directly recognizes the ac4C modification site in the 3′UTR of FOXM1 mRNA, thereby enhancing FOXM1 mRNA stability and promoting the malignant progression of CC cells. Meanwhile, overexpression of FOXM1 effectively reversed the inhibitory effects of NAT10 knockdown on proliferation, migration, and invasion of LSCC cells. Finally, animal experiments indicated that NAT10 promotes tumorigenesis in LSCC cells by upregulating FOXM1.^116^ Using inhibitors to reduce NAT10 expression in melanocytes can inhibit the expression of melanin‐stimulating genes (such as the genes encoding dopa oxidase (DCT) and tyrosinase in B16F10 melanoma cells), leading to reduced melanin synthesis and increased S‐phase cell cycle arrest, thereby inhibiting the growth and proliferation of malignant melanoma cells in vitro and in vivo.^117^ Additionally, another study discovered that using Remodelin significantly inhibited tumor growth in nude mice, as well as the in vitro growth, migration, and invasion of prostate cancer cells. Remodelin slowed DNA replication, with NAT10 regulating the expression of CDC6 and MCM7 to promote the growth of prostate cancer cells.^118^ In Clear‐cell renal cell carcinoma (ccRCC), there is a significant increase in ac4C modification and NAT10 expression levels. Elevated NAT10 promotes the proliferation and migration abilities of ccRCC cells. Further research revealed that NAT10 facilitates tumor progression and lymphangiogenesis in ccRCC by promoting the nuclear entry of the Yes1‐associated transcriptional regulator. As for downstream target genes, ANKZF1 has been identified as a functional target of NAT10, with NAT10 upregulating ANKZF1 expression through ac4C modification.^119^ Overall, these research findings indicate that ac4C plays a crucial role in regulating the malignant proliferation and growth of tumor cells.

### 5.2 NAT10 regulation of tumor cell cycle

Abnormal regulation of the cell cycle is a key mechanism in cancer progression.^120^ In normal cells, the cell cycle is tightly controlled, regulating cell growth, DNA replication, and division. However, in tumor cells, key molecules involved in cell cycle regulation, such as cyclins, cyclin‐dependent kinases (CDKs), and inhibitors, may mutate or become inactive, leading to loss of control over the cell cycle.^121^ Additionally, cell cycle dysregulation can increase genomic instability, promoting the accumulation of mutations and exacerbating tumor invasiveness and metastasis. This feature of cell cycle dysregulation highlights the importance of targeting cell cycle regulation as a critical strategy in cancer therapy. Understanding the mechanisms behind cell cycle dysregulation is essential for future cancer treatments. Early studies indicated that NAT10 primarily localizes to the midbody during late mitosis, and NAT10 depletion leads to defects in nucleolar assembly and cytokinesis, along with downregulation of α‐tubulin acetylation, resulting in G2/M arrest and mitotic block. Wang et al.^122^ found that NAT10 is significantly overexpressed in NSCLC and is associated with advanced staging of lung adenocarcinoma, shorter overall survival, and time to first progression. Increased NAT10 expression promotes NSCLC cell growth and metastasis. The upstream transcription factor c‐myc directly binds to the NAT10 promoter region, activating its expression. Knockdown of NAT10 induces cell cycle arrest at the G1 phase in lung cancer cells. The NF‐κB signaling pathway is crucial in lung cancer progression, and studies have reported that NAT10 promotes NF‐κB signaling pathway activity in lung cancer, thereby promoting lung cancer progression.^123^ Research shows that NAT10 is highly expressed in liver cancer, and higher expression correlates with shorter patient survival times. NAT10 regulates Mdm2 levels, thereby upregulating mutant p53 levels and promoting the proliferation of cells carrying p53 mutations.^124^ As a highly lethal cancer type, breast cancer has been found to exhibit abnormally high NAT10 expression. NAT10 directly acetylates MORC2, and the acetylated MORC2 binds directly to H3T11P, reducing DNA damage‐induced H3T11P, inhibiting the transcription of downstream target genes CDK1 and cyclin B1, and activating DNA damage‐induced G2 checkpoint. Furthermore, NAT10 knockdown was found to suppress the expression of melanoma‐promoting genes, causing S‐phase arrest and reduced proliferation of melanoma cells.^117^ NAT10 has also been shown to directly regulate the acetylation of CCDC84, thereby promoting ubiquitin‐mediated degradation of HsSAS‐6 and facilitating centriole duplication.^125^ Patients with HNSC have high NAT10 expression levels, which are indicative of poor prognosis. Inhibiting NAT10 expression can suppress the replication, migration, and invasion of HNSCC cell lines and induce cell cycle arrest at S/G2. Remodelin has been shown to directly inhibit NAT10 expression in HNSCC cell lines, decrease MYC expression, and upregulate LDHA expression to promote tumor spread. KIF15 has been reported to recruit NAT10, which plays a critical role in maintaining acetylated tubulin levels, ultimately regulating microtubule stability, oocyte meiosis, and cell cycle progression.^126^ Subsequent studies revealed that NAT10 is a component of the DNA replication preinitiation complex, interacting with CDC6 and replication origins to promote DNA replication.^118^ Consistent with the aforementioned, Jin et al.^51^ found that NAT10 and ac4C modification levels are significantly upregulated in CRC. High NAT10 expression is significantly associated with poor prognosis and lymphatic metastasis in patients. In vitro, NAT10 knockdown inhibits CRC cell apoptosis, promotes their proliferation, migration, and invasion, and causes G2/M phase arrest. Mechanistic studies show that NAT10 directly binds to the 3′ UTR region of KIF23 mRNA, promoting its stability through mRNA ac4C modification, thereby activating the Wnt/β‐catenin pathway, promoting β‐catenin nuclear translocation, and facilitating CRC progression.^51^ Du et al.^103^ found that NAT10 directly acetylates p53 at its K120 site, protecting it from Mdm2‐mediated degradation, stabilizing p53 expression, promoting its nuclear translocation, inhibiting apoptosis, and promoting the cell cycle. Knocking down NAT10 promotes apoptosis and differentiation in AML cells, inhibits cell proliferation, and induces cell cycle arrest. The importance of NAT10 in regulating the tumor cell cycle has garnered increasing attention. NAT10 influences the progression of the cell cycle by regulating the stability and translational efficiency of cell cycle‐related genes. Additionally, NAT10's role in cell cycle regulation extends to the modulation of cell cycle checkpoints, which ensure the integrity of cells during division. By regulating these checkpoints, NAT10 may affect the response of tumor cells to DNA damage or other stress factors, influencing cancer cell stress responses and drug resistance. In summary, NAT10 plays a biologically significant role in the regulation of the tumor cell cycle by influencing the expression of cell cycle‐related genes and the function of cell cycle checkpoints, directly participating in tumor cell proliferation and growth. Future research will further elucidate the specific mechanisms of NAT10 in cell cycle regulation and explore its potential as a therapeutic target. In summary, these findings demonstrate that ac4C is critically involved in the regulation of tumor cell cycle progression.

### 5.3 2 NAT10 regulates tumor cell metastasis and invasion

Epithelial–mesenchymal transition (EMT) is a critical process in cancer metastasis.^127^ EMT refers to the transformation of epithelial cells into mesenchymal‐like cells, enabling them to lose their fixed morphology and function and gain enhanced migratory and invasive capabilities.^128^ This transition empowers cancer cells with greater mobility, invasiveness, and resistance to apoptotic stimuli, thereby promoting tumor metastasis. During cancer metastasis, EMT not only enhances cancer cell survival and migration but also enables them to penetrate the basement membrane and invade adjacent tissues or enter the circulatory system, forming new metastatic sites far from the primary tumor.^129^ Studies have demonstrated that NAT10‐mediated ac4C modification regulates EMT and is closely linked to cancer invasiveness, metastasis, and treatment resistance. Recent research indicates that NAT10 plays a pivotal role in regulating CRC metastasis and cell motility. It was found that reduced GSK‐3β activity alters NAT10 subcellular localization, prompting NAT10 to localize to the cell membrane and cytoplasm. This change in subcellular distribution directly downregulates tubulin acetylation and decreases the expression of EMT marker E‐cadherin, ultimately promoting the invasion, migration, and in vivo metastasis of CRC cells.^55^ Consistently, a recent outstanding study has for the first time elucidated the regulatory mechanism of NAT10 in cancer metastasis. In this research, the authors utilized a combination of 2‐hydroxyisobutyrylation (Khib) proteomic analysis and CRISPR/Cas9 functional screening to identify NAT10 as a substrate of Khib modification. Further studies revealed that Khib modification at lysine 823 of NAT10 contributes to promoting its tumor metastasis function. The Khib modification of NAT10 enhanced its interaction with the deubiquitinase USP39, further increasing the stability of the NAT10 protein. NAT10 promotes metastasis by maintaining the stability of NOTCH3 mRNA in an N4‐acetylcysteine‐dependent manner. Importantly, the authors discovered a lead compound, #7586‐3507, which significantly inhibits NAT10 Khib modification and shows efficacy at low concentrations in vivo tumor models. This remarkable study further enriches the connection between RNA modification and tumor metastasis.^34^ This ultimately leads to enhanced metastasis and resistance to Lenvatinib‐induced apoptosis in ERS liver cancer cells.^130^ In lung cancer, studies have shown that NAT10 is upregulated in NSCLC tissues, cell lines, and mouse xenograft models.^131^ NAT10 expression levels are closely related to adverse clinical features such as advanced T stage, lymph node metastasis, and low overall survival. Knockdown of NAT10 inhibits proliferation, invasion, and migration, whereas overexpression of NAT10 has the opposite effect. Reducing NAT10 levels increases E‐cadherin levels and decreases N‐cadherin and vimentin expression. In contrast, recent studies have found that high NAT10 expression significantly promotes CRC metastasis and invasion. Importantly, miR‐6716‐5p, as an upstream regulatory factor, directly inhibits NAT10 expression, downregulating E‐cadherin expression and promoting CRC cell migration and invasion.^55^, ^132^ This finding seemingly contradicts earlier research^55^ (Figure 3D). Consistent with these results, another study showed that NAT10 upregulates the transcript stability of lncRNA–CTC‐490G23.2 through acetylation modification, leading to its abnormal upregulation in ESCA. CTC‐490G23.2 acts as a molecular scaffold, enhancing the binding ability of CD44 mRNA with PTBP1, regulating CD44 splicing, and promoting ESCA cancer invasion and metastasis^77^ (Figure 3E). Interestingly, Remodelin inhibits NSCLC proliferation, invasion, and migration by inhibiting NAT10 via the EMT pathway.^131^ Key molecules in EMT, such as transcription factors Twist, Snail, and Slug, regulate this process by orchestrating cytoskeletal reorganization and disrupting intercellular junctions, thereby facilitating cancer cell metastasis. Additionally, NAT10 knockdown was found to significantly upregulate the expression of the EMT marker cadherin 1 (E‐cadherin) and downregulate vimentin expression in hepatocellular carcinoma cells, inhibiting invasion and migration in vitro.^132^ Moreover, altered subcellular localization of NAT10 was shown to significantly enhance the invasive and migratory abilities of hepatocellular carcinoma cells.^133^ In prostate cancer, NAT10 expression has been found to be significantly elevated. Elevated NAT10 expression significantly promotes cell cycle arrest and EMT in prostate cancer cells, thus advancing malignant progression. Mechanistic studies indicate that NAT10 enhances the mRNA stability of HMGA1 and KRT8 through acetylation modification, thereby increasing their protein levels, which in turn regulate cell cycle progression and EMT^134^ (Figure 3F). In breast cancer cells, NAT10 knockdown reversed docetaxel‐induced EMT and restored sensitivity to docetaxel, as evidenced by upregulated CDH1 (E‐cadherin) expression and downregulated VIM (vimentin) expression.^135^ Recent studies have found that NAT10 is abnormally highly expressed in gastric cancer tissues, significantly promoting the metastasis and invasion of gastric cancer cells. Further research found that high NAT10 expression promotes the expression of IM and MMP2 through RNA acetylation modification, ultimately promoting EMT in gastric cancer.^136^ Meanwhile, COL5A1, another EMT‐related downstream target gene of NAT10, is regulated by NAT10. Molecular mechanism studies show that NAT10 directly binds to the 3′ UTR region of COL5A1 mRNA in an ac4C modification‐dependent manner, promoting COL5A1 expression and thus promoting gastric cancer cell metastasis and EMT^137^ (Figure 3H). Moreover, knocking down NAT10 significantly inhibits the proliferation, invasion, and migration of cervical cancer cells. This is achieved through NAT10's promotion of ac4C modification and stabilization of HNRNPUL1 mRNA, leading to upregulated HNRNPUL1 expression. Loss of HNRNPUL1 suppresses the division, invasion, and migration of cervical cancer cells. This study indicates that NAT10 enhances the stability of HNRNPUL1 mRNA via ac4C modification, thereby promoting the progression of cervical cancer.^138^ NAT10 plays a crucial role in regulating EMT and cancer metastasis in tumor cells. Through its regulation of RNA ac4C, NAT10 directly influences the expression of key genes involved in EMT. NAT10 modulates the acetylation of transcription factors related to EMT, such as Twist, Snail, and ZEB1, which are key drivers of EMT. By modifying the mRNA of these transcription factors, NAT10 affects their stability and translational efficiency, thereby regulating the onset and progression of EMT. Additionally, NAT10 promotes cytoskeletal remodeling and cell motility, key aspects of cancer metastasis, by regulating genes involved in cytoskeletal dynamics. NAT10 also influences EMT and tumor metastasis through its regulation of cell adhesion molecules, such as E‐cadherin and vimentin. Abnormal expression of NAT10 disrupts the normal function of these adhesion molecules, enhancing the detachment and migration of cancer cells, and ultimately promoting tumor invasion and metastasis. In summary, NAT10 plays a significant role in regulating EMT and cancer metastasis by influencing the expression of EMT‐related genes, modulating the cytoskeleton, and regulating the function of cell adhesion molecules. It directly contributes to tumor cell invasion and metastasis. Future research will further elucidate the specific mechanisms of NAT10 in EMT and cancer metastasis and explore its potential as a therapeutic target to curb tumor spread and metastasis. Collectively, the evidence suggests that ac4C plays an essential role in regulating tumor cell metastasis and invasion.

### 5.4 NAT10 regulation of tumor cell death

Dysregulation of tumor cell death is a key factor in cancer progression.^139^ In normal tissues, cell death, particularly programmed cell death such as apoptosis, is an essential mechanism for maintaining tissue homeostasis.^140^ However, tumor cells often evade immune clearance and apoptotic signals through various mechanisms, such as mutations that inhibit apoptosis‐related proteins (e.g., p53) or activate antiapoptotic pathways (e.g., the Bcl‐2 family).^141^ Studies indicate that NAT10 mediates the acetylation of ferroptosis suppressor protein 1 (FSP1), the FSP1–CoQ10–NAD(P)H axis, and the vitamin K redox cycle^142^ (Figure 4A). NAT10 regulates FSP1 expression through ac4C acetylation, increasing reactive oxygen species (ROS), ferrous ion levels, MDA, mitochondrial matrix condensation, and cristae levels, inhibiting ferroptosis in colon cancer cells. In breast cancer cells with NAT10 knockdown or KO, ac4C levels decrease, leading to the downregulation of several key ferroptosis genes, including SLC7A11, GCLC, MAP1LC3A, and SLC39A8^143^ (Figure 4A). Furthermore, Zhang et al.^144^ confirmed that MORC2 enhances PARP1 acetylation at K949 through NAT10, stabilizing PARP1 and protecting against DNA damage (Figure 4B). This resistance to apoptosis enables tumor cells to survive in a hostile microenvironment, fueling cancer progression. In addition to apoptosis, other forms of cell death regulation, such as autophagic cell death and necrosis, are also closely linked to tumor survival and treatment resistance. The ability of tumor cells to evade death not only promotes tumor growth and progression but also impairs therapeutic efficacy, posing a major challenge in cancer treatment.^145^ Another study showed that abnormally overexpressed NAT10 in ESCA is significantly negatively correlated with tumor diameter and overall survival. Knockdown of NAT10 significantly promotes ESCA cell apoptosis and inhibits cell proliferation. Using siRNA to further inhibit NAT10 expression upregulates Caspase3.^146^ Knocking down NAT10 decreases RNA acetylation levels, while overexpression promotes RNA acetylation. NAT10 directly binds to CEP170, enhancing its acetylation level and promoting its protein translation, thereby facilitating the progression of multiple myeloma (MM)^147^ (Figure 4E). Studies have shown that NAD+ increases the expression levels of NAT10, enhancing PARP1 acetylation and promoting human glioma cell death^148^ (Figure 4F). Ferroptosis is a novel form of programmed cell death characterized by iron overload and lipid peroxidation accumulation within cells.^149^, ^150^, ^151^ Unlike traditional forms of cell death such as apoptosis, necrosis, and autophagy, ferroptosis is primarily caused by iron‐dependent reactions, leading to increased ROS and lipid peroxidation.^152^, ^153^, ^154^, ^155^ Oh et al.^117^ used siRNA to inhibit NAT10 expression in human and mouse melanoma cells, resulting in S‐phase cell cycle arrest, with the mechanism involving NAT10 regulation of the p21/CDK2/cyclin D1 axis (Figure 4G). Ferroptosis plays a critical role in various pathological processes, including neurodegenerative diseases, cancer, and cardiovascular diseases.^156^, ^157^ Studies suggest that modulating the molecules and signaling pathways involved in ferroptosis can significantly influence disease progression and therapeutic outcomes.^143^, ^158^, ^159^ NAT10‐mediated ac4C acetylation plays a key role in regulating ferroptosis. NAT10 is a rRNA acetyltransferase that catalyzes the acetylation of nitrogen at the 4‐position (ac4C), affecting RNA stability and translation efficiency. During ferroptosis, NAT10‐mediated ac4C modifications can regulate the expression of key genes, promoting or inhibiting the metabolism and balance of intracellular iron ions. This modification influences the expression levels of iron metabolism‐related genes and antioxidant genes, regulating ROS generation and lipid peroxidation, thereby playing an important role in ferroptosis. Research suggests that regulating NAT10 activity or ac4C acetylation levels could provide new targets and strategies for treating ferroptosis‐related diseases.^160^ These results suggest that NAT10 and its mediated ac4C acetylation modifications may play a critical role in regulating ferroptosis in tumor cells. Targeting this mechanism could potentially provide new therapeutic strategies for treating cancer and other diseases. Recent studies have primarily focused on the role and mechanisms of NAT10 in ferroptosis, with a particular emphasis on NAT10‐mediated ac4C acetylation's impact on the expression of key genes involved in ferroptosis resistance. As research on cell death deepens, it is anticipated that the functions of NAT10 in other forms of cell death, such as pyroptosis and cuproptosis, will also be progressively elucidated. Given the crucial role of cell death in chemoresistance in cancer, targeting NAT10 to address chemoresistance induced by ferroptosis presents a potential therapeutic strategy. In general, these research findings highlight the significant role of ac4C in controlling tumor cell death and cancer progression.

### 5.5 Regulation of tumor cell drug resistance by NAT10

Drug resistance in tumor cells is a significant cause of cancer progression and treatment failure.^161^ During cancer therapy, tumor cells often acquire resistance to chemotherapy, radiotherapy, or targeted therapies through various mechanisms. These mechanisms include overexpression of drug efflux pumps,^162^ mutations or loss of drug target genes, enhanced DNA repair pathways, and activation of antiapoptotic pathways. These alterations enable tumor cells to survive treatment and continue proliferating. Additionally, tumor cell drug resistance may be exacerbated by complex signaling and heterogeneity within the tumor microenvironment (TME), such as the presence of cancer stem cells and interactions among tumor cells, allowing resistant cells to evade treatment and contributing to cancer relapse and metastasis.^163^ The emergence of drug resistance severely limits the effectiveness of existing treatments, making cancer control and cure increasingly challenging. Recent research indicates that abnormal expression of NAT10 may be closely associated with drug resistance in tumor cells. A recent study showed that inhibiting NAT10 expression suppresses the proliferation and invasion of breast cancer cells. Mechanistic studies indicated that NAT10 directly binds to the transcripts of ABC transporter, multidrug resistance protein 1, and breast cancer resistance protein, regulating their RNA acetylation levels, maintaining their stability, and promoting breast cancer cell drug resistance and progression.^164^ NAT10 directly interacts with PARP1, promoting the acetylation of PARP1 transcripts, conferring resistance to platinum‐based drugs, regulating the breast cancer EMT process, and promoting doxorubicin resistance.^135^ Due to the heterogeneity of tumors, the mechanisms of tumor resistance are complex and diverse. However, NAT10‐mediated ac4C acetylation can regulate the drug resistance of certain tumors, providing a basis for targeting NAT10‐mediated ac4C acetylation to improve tumor resistance. In addition, NAT10 has been reported to play a significant role in cancer drug resistance. For instance, in bladder cancer, NAT10‐mediated Ac4C modification significantly enhances the mRNA stability of genes associated with DNA damage repair, thereby promoting cisplatin resistance. Interestingly, cisplatin directly induces activation of the NF‐κB signaling pathway, increasing NAT10 transcription levels and leading to its aberrant upregulation. Elevated NAT10 expression can maintain the stability of AHNAK mRNA, protecting it from degradation by nucleases and ultimately promoting AHNAK‐mediated DNA damage repair.^165^ In liver cancer, NAT10 directly upregulates the ac4C modification levels of HSP90AA1 mRNA, enhancing its stability and improving its antiapoptotic capacity against lenvatinib.^130^ NAT10‐mediated ac4C acetylation is crucial in combating multidrug resistance. Future extensive research on NAT10's role in tumor multidrug resistance mechanisms could lead to the development of new drugs targeting NAT10, expanding treatment options for drug‐resistant cancers. Wu et al.^135^ demonstrate that siRNA targeting NAT10 or Remodelin can significantly reverse doxorubicin resistance in breast cancer cell lines, indicating that NAT10 plays a role in the development of doxorubicin resistance. The combined use of Remodelin and doxorubicin shows a synergistic effect, significantly inhibiting tumor growth, suggesting that this combination may enhance chemotherapy efficacy. Understanding the complex interactions between NAT10 and chemoresistance could lead to the development of new strategies to address treatment resistance and improve patient prognosis. On the whole, the evidence underscores the critical function of ac4C in mediating tumor cell drug resistance.

### 5.6 Regulation of tumor cell metabolism by NAT10

Aberrant tumor cell metabolism is a significant hallmark of cancer progression. Unlike normal cells, tumor cells often rely on the “Warburg effect,” a metabolic reprogramming that not only supports rapid proliferation but also provides the metabolic intermediates necessary for synthesizing macromolecules.^166^ Additionally, tumor cells remodel metabolic pathways involving glucose, lipids, and amino acids to adapt to stress conditions such as hypoxia and nutrient deprivation in the microenvironment, thereby enhancing their survival and invasiveness.^167^ NAT10 has been reported to regulate the expression of various target genes through Ac4C modification, participating in different aspects of cancer metabolism, including glucose metabolism, lipid metabolism, and amino acid metabolism. Moreover, many key metabolic genes, such as ACSL1 and ACAT1, are regulated by NAT10 through ac4C acetylation, participating in fatty acid metabolism. The loss or mutation of these genes significantly inhibits fatty acid metabolism, downregulating lipid content, triglyceride, and total cholesterol levels^168^ (Figure 4C).Recent studies have found that HIF‐1 can directly transcriptionally activate NAT10, upregulating its expression, which in turn promotes NAT10‐mediated ac4C acetylation of SEPT9 mRNA, activates the HIF‐1 pathway, regulates glucose metabolism reprogramming in gastric cancer, and forms a NAT10/SEPT9/HIF‐1 positive feedback loop, promoting glycolysis‐dependent and malignant development of gastric cancer^84^ (Figure 4D). In tumor cells, NAT10‐mediated ac4C acetylation can promote glycolysis and lipid metabolism pathways, providing the energy and biosynthetic precursors needed for rapid growth.^34^ Its acetylation function plays a crucial role in tumor metabolic reprogramming. Therefore, in‐depth research on the mechanisms of NAT10‐mediated ac4C acetylation in tumor metabolism can help reveal new aspects of tumor metabolic regulation and provide new targets and strategies for cancer treatment. A recent study found that the key ac4C acetylation enzyme NAT10 is highly expressed in human osteosarcoma tissues. Knocking down NAT10 significantly increases m6A levels and markedly inhibits the growth, migration, and invasion of osteosarcoma cells. Additionally, reducing NAT10 expression inhibits the stability and translation of YTHDC1 mRNA, increases glucose uptake and lactate production, and decreases pyruvate levels. YTHDC1 further recognizes differential m6A sites on key glycolytic enzymes PFKM and LDHA in an m6A‐dependent manner, increasing their mRNA stability and thus inhibiting glycolysis. This study reveals the critical role of the NAT10/ac4C–YTHDC1/m6A–LDHA/PFKM signaling axis in osteosarcoma.^79^ In cervical cancer tissues, NAT10 expression is significantly increased, which is associated with poor clinical prognosis. Further research reveals that HOXC8 activates NAT10 by binding to its promoter, thereby stimulating ac4C modification of FOXP1 mRNA and enhancing its translation efficiency. This leads to the induction of GLUT4 and KHK expression. Additionally, the activity of the NAT10/ac4C/FOXP1 axis results in increased glycolysis and sustained lactate secretion in CCa cells. The lactate‐rich TME further enhances the immunosuppressive properties of tumor‐infiltrating regulatory T cells (Tregs)^169^ (Figure 3I). Despite numerous studies elucidating the molecular mechanisms of cellular metabolism, the complexity and heterogeneity of cancer metabolism reflect the diversity and challenges inherent in cancer biology. The metabolic processes of cancer cells differ markedly from those of normal cells; these pathways are not only altered to support tumor growth and metastasis but also exhibit considerable individual variability. Furthermore, cancer cell metabolism can be regulated through various mechanisms, including genetic mutations, activation or inhibition of signaling pathways, and changes in the expression of metabolic enzymes. These alterations may result in differences in metabolic demands and adaptive capacities among cancer cells, thereby influencing their response to treatment and prognosis. Additionally, the TME significantly impacts cancer metabolism.^170^ The types of cells present in the TME, blood supply, and immune cell presence all affect the metabolic state of cancer cells. Different microenvironmental conditions may lead cancer cells to adopt distinct metabolic strategies, further increasing the heterogeneity of cancer metabolism.^171^ In summary, the complexity and heterogeneity of cancer metabolism pose significant challenges for cancer research and treatment. A deeper understanding of these metabolic differences could provide crucial insights for developing personalized treatment strategies and more effective cancer therapies. Therefore, therapeutic design must consider the metabolic susceptibilities of both cancer and noncancer cells within the tumor immune microenvironment. Subsequent research will explore how various metabolites influence NAT10‐mediated ac4C acetylation, ultimately promoting malignant tumor progression. Overall, these studies reveal the pivotal role of ac4C in regulating tumor cell metabolism.

### 5.7 Regulation of Tumor Immunity by NAT10

**FIGURE 5:**
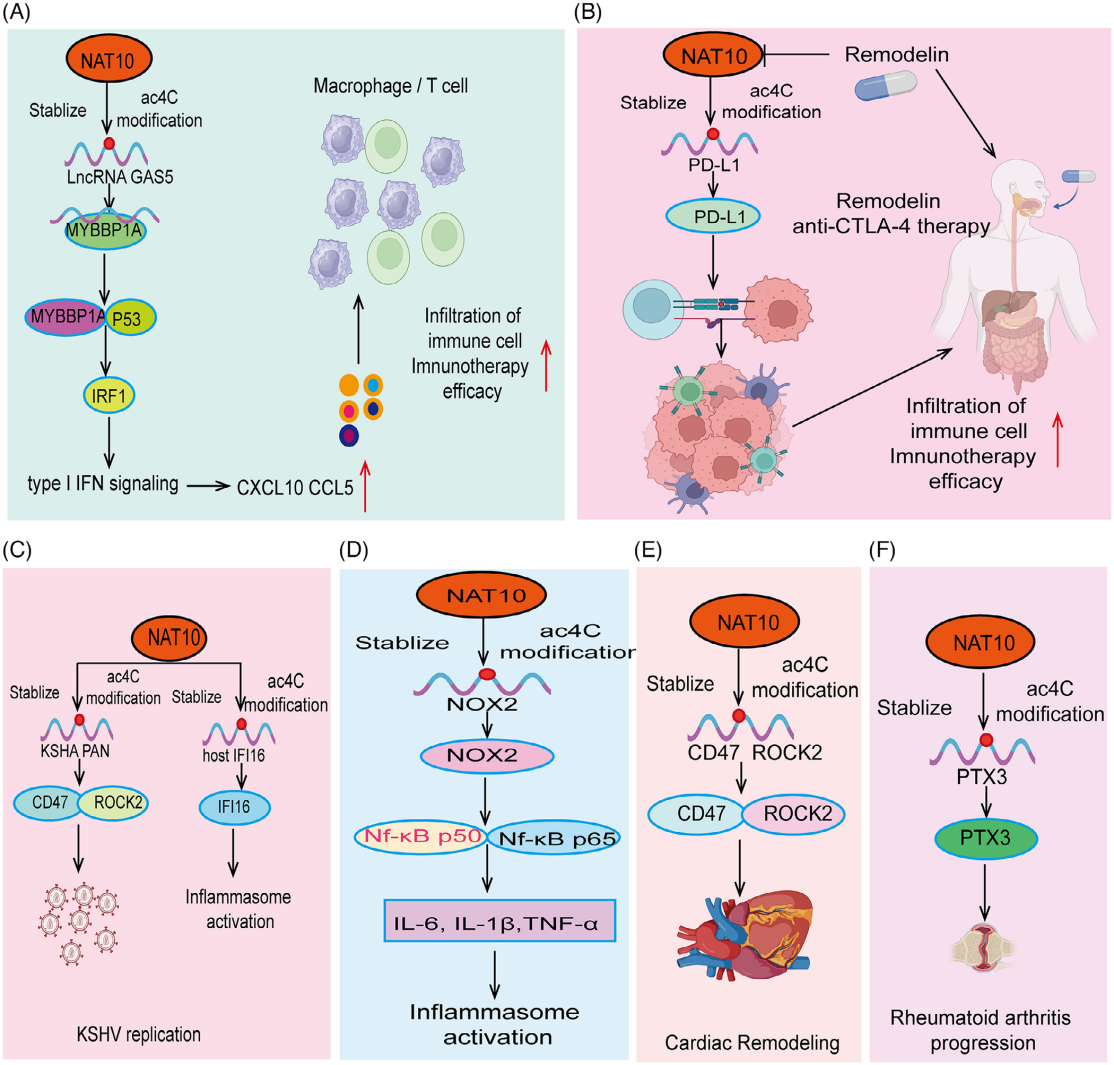
Functions and mechanisms of NAT10‐mediated AC4C modification in immune regulation and other diseases. (A and B) The role of NAT10‐mediated AC4C modification in immunotherapy. (C–F) The involvement of NAT10‐mediated AC4C modification in (C) KSHV replication, (D) inflammatory activation, (E) cardiovascular diseases, and (F) arthritis. CCL5, C‐C motif chemokine ligand 5; CTLA4, cytotoxic T‐lymphocyte associated protein 4; CD47, cluster of differentiation 47; ROCK2, Rho‐associated coiled‐coil containing protein kinase 2; PTX3, pentraxin 3.

Tumor immune evasion is a key mechanism in cancer progression.^172^ Under normal circumstances, the immune system can recognize and eliminate aberrant tumor cells, using immune surveillance mechanisms to prevent cancer onset and spread. However, tumor cells employ various strategies to evade immune system attacks, including downregulation of major histocompatibility complex (MHC) molecule expression,^173^ secretion of immunosuppressive cytokines (e.g., TGF‐β, IL‐10), induction of Tregs and myeloid‐derived suppressor cells (MDSCs), and suppression of effector T cell functions. These immunosuppressive factors in the TME collectively weaken antitumor immune responses, allowing tumor cells to survive, proliferate, and metastasize. Additionally, tumor cells inhibit T cell activity through immune checkpoint pathways such as PD‐1/PD‐L1, further promoting cancer progression.^174^ Immune evasion enables tumors to escape immune system control, thereby facilitating cancer development and progression, and providing important targets for immunotherapy.^175^ Research has reported that the expression of many immune checkpoint molecules is also regulated by NAT10‐mediated Ac4C modification. Moreover, the infiltration levels of various immune cells are related to NAT10. Recent studies have shown that NAT10 stabilizes GAS5 transcripts through acetylation modifications, and the upregulated GAS5 is positively correlated with macrophage and T‐cell infiltration in NSCLC. Increased GAS5 expression promotes the recruitment of macrophages and T cells both in vitro and in vivo. Tumor cell‐derived GAS5 can activate type I interferon signaling through the MYBBP1A–p53/IRF1 axis, promoting immune cell infiltration and potentially being associated with the efficacy of immunotherapy, thus inhibiting the progression of NSCLC^122^ (Figures 3G and 5A). Studies have demonstrated that high expression of NAT10 promotes the acetylation of NPM1, which further enhances the stability of its transcripts, thereby upregulating the transcription of the immune checkpoint PD‐L1. The use of the NAT10 inhibitor Remodelin effectively reduces the acetylation of NPM1, leading to decreased PD‐L1 expression. In vivo experiments have shown that the combination of Remodelin with anti‐CTLA‐4 therapy is more effective than using either therapy alone (Figure 5B). These results indicate that RNA acetylation modifications are crucial in tumor immunotherapy.^176^

Immunotherapy is a treatment method that utilizes the body's own immune system to fight diseases. The basic principle is to enhance, regulate, or repair the immune system's functions to more effectively recognize and attack abnormal cells or pathogens.^177^, ^178^ Immunotherapy is widely used to treat various diseases, especially different types of cancer, by inhibiting inhibitory signal molecules (immune checkpoints) and enhancing the immune cells’ ability to recognize and attack tumor cells, such as PD‐1 inhibitors and CTLA‐4 inhibitors.^179^, ^180^, ^181^ The development of immunotherapy has brought new hope for treating diseases that are difficult to control with traditional therapies, but it also faces challenges such as immune‐related adverse reactions and the sustainability of therapeutic effects.^182^, ^183^ As a primary treatment method for cervical cancer, the development of immunotherapy is mainly attributed to the breakthrough clinical achievements of immune checkpoint inhibitors (ICIs) such as PD‐1/PD‐L1 antibodies. Recent research on NAT10‐catalyzed ac4C modifications in immune dysregulation and tumor immunotherapy response has found that NAT10 is significantly upregulated in cervical cancer and is associated with poorer survival in cervical cancer patients. Subsequent studies have shown that the oncogenic transcription factor HOXC8 directly binds to the promoter region of NAT10, transcriptionally activating its expression, thereby upregulating NAT10 protein levels. NAT10 upregulates the ac4C modification of FOXP1 mRNA and increases its translation efficiency, ultimately upregulating the expression of GLUT4 and KHK, promoting increased glycolysis and sustained lactate secretion in cervical cancer cells.^169^ Interestingly, the NAT10/ac4C/FOXP1 axis activity in the lactate‐rich TME further enhances the immunosuppressive properties of tumor‐infiltrating Tregs.^169^ In vivo experiments have shown that knocking down NAT10 significantly improves the efficacy of PD‐L1 blockade‐mediated tumor regression in vivo. This interesting study reveals the oncogenic role of NAT10‐mediated ac4C in the crosstalk between glycolysis regulation and immunosuppression in cancer cells.^169^ These results suggest that NAT10 plays an important role in regulating the immune microenvironment, warranting further in‐depth exploration in future research. Similar research indicates that NAT10, the only known “writer” of ac4C mRNA modification, is highly expressed in HNSC patients with lymph node metastasis, and this elevated expression is closely associated with poorer prognosis in patients. The transcription factor NRF1 positively regulates NAT10 expression. Knockdown of NAT10 significantly inhibits cell migration in mice. Mechanistic studies reveal that NAT10 induces ac4C modification of GLMP mRNA and stabilizes its expression, thereby activating the MAPK/ERK signaling pathway and reshaping the TME, including angiogenesis, recruitment of CD8+ T cells and Tregs, thereby promoting HNSC progression.^184^ The greatest challenge in tumor immune therapy is the unique and complex immune microenvironment of tumors, which necessitates a thorough understanding of its mechanisms to enable normal immune cells to effectively target and eliminate tumors. The role of NAT10 in the tumor immune microenvironment remains unclear, and future research on NAT10's function in this context will be crucial for revealing mechanisms of tumor immune evasion. As a significant epigenetic modifier enzyme, NAT10 regulates various biological processes through RNA ac4C acetylation, potentially playing a key role in modulating the TME. First, research will likely focus on how NAT10 regulates immune cell functions. By modulating ac4C acetylation levels in immune cells, NAT10 may impact immune cell activity, proliferation, and cytokine secretion, thereby altering the intensity and nature of immune responses within the TME. For instance, variations in NAT10 could influence the expression of immune checkpoint molecules, affecting immune evasion and tumor progression. Second, NAT10's role within tumor cells will also be of interest. NAT10 might affect tumor cells' responses to immune attacks by regulating RNA acetylation, influencing antigen presentation, or modifying tumor cell metabolic features, which could, in turn, impact the construction and function of the immune microenvironment. Additionally, interactions between NAT10 and other components of the TME, such as cancer‐associated fibroblasts, endothelial cells, and immunosuppressive cells, will be a research focus. These interactions might influence tumor growth and metastasis through NAT10 regulation and could provide new targets for novel immune therapies. In summary, future research on NAT10 in the tumor immune microenvironment will help elucidate its role in tumor immune escape and therapy resistance, advancing the field of immunotherapy and offering new strategies for personalized treatment. Further studies are expected to enhance understanding of NAT10's functions and develop more effective intervention strategies in tumor immune therapy. In summary, these findings point to the critical role of ac4C in modulating the tumor immune microenvironment.

## 6 ROLE OF NAT10 IN OTHER DISEASES

Numerous studies have reported on the role of Ac4C in normal physiological processes and tumor progression. However, research on Ac4C in other nontumor diseases, particularly in vascular diseases, metabolic disorders, neurological diseases, and autoimmune diseases, is still in its early stages. Therefore, we summarize the current research progress on Ac4C modifications in these diseases.

### 6.1 Role of NAT10 in systemic lupus erythematosus

Epigenetic abnormalities can disrupt the immune microenvironment, altering the function of various immune cells and impacting the progression of autoimmune diseases.^185^ Studies have shown that NAT10 and global ac4C levels are downregulated in CD4+ T cells from patients with systemic lupus erythematosus (SLE), with abundant ac4C peaks in mRNA CDSs and 3′ UTR regions, indicating dysregulation of ac4C modifications in SLE.^186^ Conversely, in HeLa cells, high densities of ac4C sites are observed around translation initiation sites (5′ UTR and CDS), suggesting that the localization of ac4C modifications may be disease‐specific,^26^ In fact, ac4C peaks in CD4+ T cells from SLE patients could serve as significant targets. In summary, these studies highlight the crucial role of ac4C modification in SLE.

### 6.2 Role of NAT10 in infectious diseases

Recent studies have shown that during alphavirus infection, the expression of NAT10 is significantly upregulated, and the level of ac4C modification is elevated in host cells. Depletion of NAT10 or inhibition of its N‐acetyltransferase activity markedly suppresses alphavirus replication. Further investigation revealed that NAT10 promotes viral replication by stabilizing the mRNA of LY6E, a well‐known interferon‐stimulated gene, through ac4C modification.^187^ Furthermore, high levels of NAT10 are present in HIV‐1, and reducing NAT10 expression significantly downregulates ac4C levels in viral RNA and HIV‐1‐associated marker gene expression, reducing viral RNA stability and ultimately inhibiting HIV replication. The small molecule inhibitor of NAT10, Remodelin, can significantly suppress HIV‐1 replication without affecting normal cell function, suggesting that targeting ac4C may be a potential strategy for antiviral drug development.^188^ Similarly, a reduction in NAT10 has been observed in the influenza A virus, where NAT10 interacts with the M1 viral protein to promote ac4C modification levels on viral RNA.^189^ Consistent with previous findings, recent studies have identified that NAT10 mediates ac4C modification on the long noncoding RNA (PAN) encoded by Kaposi's sarcoma‐associated herpesvirus (KSHV), which triggers viral lytic reactivation from latency. Under KSHV infection conditions, the knockdown of NAT10 expression results in decreased ac4C modification on PAN RNA and reduced RNA stability, thereby inhibiting the KSHV activation process. Additionally, PAN ac4C modification promotes the interaction between NAT10 and IFI16 mRNA, enhancing its ac4C acetylation, mRNA stability, and translation, ultimately leading to inflammasome activation^190^ (Figure 5C). Research reports that NAT10 maintains the stability of NOX2 through acetylation modification, thereby activating the ROS–NF‐κB pathway to regulate LPS‐induced inflammatory activation in macrophages and promote the progression of periodontitis (Figure 5D). In summary, these studies highlight the crucial role of ac4C modification in the viral life cycle and antiviral immunity.

### 6.3 Role of NAT10 in metabolic diseases

Furman et al.^191^ found that increased levels of ac4C and adenine in rats are associated with hypertension. Ac4C and adenine jointly promote the expression of the NLR family NLRC4 gene, activate the NLRC4 inflammasome, and increase IL‐1β production, which in turn activates platelets and leukocytes, leading to elevated blood pressure. They hypothesize that increased oxidative stress in the elderly accelerates tRNA degradation and raises ac4C levels. Additionally, ac4C has been linked to gestational diabetes mellitus (GDM).^191^ Law et al.^192^ analyzed urine samples from 27 Chinese GDM patients and 34 normal pregnant women, finding increased levels of tryptophan metabolites and purine nucleosides in GDM patients’ urine, as well as elevated levels of methylated or modified bases, including ac4C. Niwa et al.^193^ investigated the relationship between uremia and human nucleoside metabolism by comparing nucleoside metabolite levels in serum and urine samples from multiple patient groups, revealing significantly reduced ac4C levels in the urine of uremic patients with chronic kidney failure who did not require dialysis. These findings suggest altered RNA metabolism and abnormal accumulation of modified nucleosides (e.g., ac4C) in uremic patients.^193^ In summary, these studies highlight the crucial role of ac4C modification in metabolic diseases.

### 6.4 Role of NAT10 in cardiovascular diseases

A recent study indicated that compared with normal control samples, the expression levels of NAT10 and ac4C acetylation modification were abnormally upregulated in patients with dilated cardiomyopathy. In vitro and in vivo experiments showed that knocking out or inhibiting NAT10 expression significantly alleviated heart failure symptoms. Mechanistic studies revealed that NAT10 regulates the transcript stability of CD47 and ROCK2 through ac4C acetylation, thereby activating downstream signaling pathways and regulating cardiac remodeling mechanisms in heart failure^194^ (Figure 5E). In summary, these studies highlight the crucial role of ac4C modification in cardiovascular diseases.

### 6.5 Role of NAT10 in autoimmune diseases

To explore the relationship between progressive relapsing multiple sclerosis (PRMS) and nucleoside metabolites, Bhargava et al.^195^ analyzed plasma metabolites from 18 healthy individuals and 18 patients. They found that 58 metabolites, including ac4C, were significantly elevated in multiple sclerosis patients compared with healthy controls. Moreover, there was no significant difference in ac4C levels before and after dimethyl fumarate (DF) treatment in multiple sclerosis patients.^195^ Rheumatoid arthritis (RA) is a chronic autoimmune disease that mainly affects the joints but can also involve multiple organs and systems.^196^, ^197^ It is characterized by chronic inflammation of the synovial membrane, leading to joint swelling, pain, stiffness, and functional impairment. As the disease progresses, irreversible damage such as bone erosion and joint deformity may occur.^198^ A recent study found that NAT10 and ac4C levels were significantly upregulated in fibroblast‐like synoviocytes from RA patients. Knocking out NAT10 or using specific inhibitors could inhibit the migration and invasion of RA FLSs. In synovial tissue, NAT10 expression was positively correlated with the infiltration of various immune cells. Inhibiting NAT10 expression in mice alleviated the severity of arthritis in CIA and DTHA mice and CIA rats. Mechanistic studies demonstrated that NAT10 regulates the acetylation of PTX3 mRNA, providing stability and translation of PTX3, thereby promoting the invasion and function of fibroblast‐like synoviocytes^199^ (Figure 5F). In summary, these studies highlight the crucial role of ac4C modification in autoimmune diseases.

### 6.6 Role of NAT10 in neurodegenerative diseases

HGPS is a severe, incurable premature aging disorder caused by mutations in the lamin A gene (LMNA). LMNA encodes lamin A and lamin C, and mutations can lead to abnormal cleavage of lamin A and lamin C precursors, resulting in the production of the progerin protein.^200^ The pathogenic progerin disrupts nuclear shape and chromatin organization, causing cellular distortions, irregular nuclear morphology, DNA damage, and interference with cell signaling pathways, leading to HGPS.^98^ NAT10 plays a role in HGPS pathogenesis. Inhibition of NAT10 expression can restore normal nuclear morphology in HGPS patient cells through microtubule deacetylation and cytoskeletal reorganization. Research has identified acetylation sites on lamin A, associated with chromatin anchoring.^201^ However, studies linking NAT10 with lamin A acetylation are limited. Larrieu et al.^96^ discovered that LMNA mutations in HGPS patients downregulate nuclear structural proteins lamin A and C, resulting in nuclear and chromatin abnormalities. The use of NAT10 inhibitor Remodelin can inhibit NAT10 activity, restore microtubule regeneration in LMNA‐deficient cells, and reduce microtubule membrane anchoring in HGPS patient cells, thus helping to maintain normal nuclear and chromosomal structure and achieve therapeutic goals for HGPS.^96^ In 2018, another study found that fibroblasts from HGPS patients exhibited nuclear‐cytoplasmic transport defects, and NAT10 inhibition improved the HGPS phenotype by rebalancing the nuclear‐cytoplasmic ratio of TNPO1, suggesting that NAT10 inhibitors might be a new strategy for treating HGPS.^99^ In summary, these studies highlight the crucial role of ac4C modification in neurodegenerative diseases.

## 7 ROLE OF NAT10 IN TUMOR PROGNOSTIC MARKERS AND CANCER THERAPY

### 7.1 NAT10 as a tumor prognostic marker

NAT10, as a critical epitranscriptional regulatory factor, has garnered significant attention in recent years for its role in tumor prognosis assessment. By modulating ac4C modifications on RNA, NAT10 influences mRNA stability and translation, playing a pivotal role in cancer development and treatment. Studies have shown that abnormal NAT10 expression is closely associated with the prognosis of various tumors, with high levels of NAT10 often correlating with poor outcomes. This suggests that NAT10 may serve as an important biomarker for evaluating tumor progression and treatment response. Further investigation into the role of NAT10 in tumor biology could enable more precise prognostic assessments for patients and help optimize therapeutic strategies, ultimately improving clinical outcomes. For example, In lung cancer, studies have shown that NAT10 is upregulated in NSCLC tissues, cell lines, and mouse xenograft models.^131^ NAT10 expression levels are closely related to adverse clinical features such as advanced T stage, lymph node metastasis, and low overall survival. Wang et al.^122^ found that NAT10 is significantly overexpressed in NSCLC and is associated with advanced staging of lung adenocarcinoma, shorter overall survival, and time to first progression. Studies have shown that NAT10 is significantly upregulated in ESCA. Patients with high NAT10 expression have significantly shorter survival times. Another study showed that abnormally overexpressed NAT10 in ESCA is significantly negatively correlated with tumor diameter and overall survival. Further research indicates that high NAT10 expression is negatively correlated with overall survival in CRC patients. Consistent with the aforementioned, Sun et al.^51^ found that NAT10 and ac4C modification levels are significantly upregulated in CRC. High NAT10 expression is significantly associated with poor prognosis and lymphatic metastasis in patients. Research shows that found that NAT10 is located in the nucleus/nucleolus and is significantly more highly expressed in HCC than in the control group. Cox regression analysis (both univariate and multivariate) demonstrated that NAT10 expression is an independent prognostic factor for HCC patient survival.^202^ Additionally, a recent study revealed that high NAT10 expression is associated with increased liver cancer risk and poor prognosis. Another study on glioma showed that NAT10 could serve as a significant and effective prognostic factor in glioma.^203^ NAT10 is abnormally overexpressed in MM and is associated with poor clinical outcomes in patients. As a hematological malignancy, AML patients have higher NAT10 expression. Compared with patients with low NAT10 expression, those with high NAT10 expression in AML have significantly shorter survival times.^204^ Another similar study indicated that NAT10 expression is higher in NPM1 mutant patients than in NPM1‐wt patients and is negatively correlated with progression‐free survival and overall survival.^204^ Patients with HNSC have high NAT10 expression levels, which are indicative of poor prognosis. Consistent with our analysis of the TCGA database results for HNSC, NAT10 is a promising predictive biomarker for HNSCC patients.^205^ Similar research indicates that NAT10, the only known “writer” of ac4C mRNA modification, is highly expressed in HNSC patients with lymph node metastasis, and this elevated expression is closely associated with poorer prognosis in patients. High NAT10 expression is observed in BLCA and is significantly associated with lymph node and distant metastasis and poor prognosis.^33^ In prostate cancer, NAT10 expression has been found to be significantly elevated. High NAT10 expression is closely associated with patients’ pathological grade, clinical stage, Gleason score, and TN stage.^134^ In cervical cancer tissues, NAT10 expression is significantly increased, which is associated with poor clinical prognosis.^169^ In ovarian cancer, a significant upregulation of NAT10 has been observed, and this is notably associated with patient prognosis.^206^

Overall, these reports highlight the critical role of NAT10 as a prognostic and diagnostic biomarker. The detection of NAT10 can aid in assessing disease progression and treatment response in patients, with particular significance in the early diagnosis of tumors. However, the limitations of NAT10 as a biomarker should not be overlooked. First, its expression levels may exhibit heterogeneity across different tumor types and subtypes, restricting its broad applicability. Second, the functional mechanisms of NAT10 remain incompletely understood, and there is a lack of sufficient studies to support its full clinical implementation. Therefore, further research is required to validate its biomarker functions and explore its potential in combination with other biomarkers to improve diagnostic accuracy and prognostic prediction.

### 7.2 The application of targeting NAT10 in cancer therapy

Given the crucial role of NAT10 and its mediated RNA ac4C modification in disease progression, particularly in tumors, extensive research has been conducted on using specific small molecule inhibitors of NAT10 for therapeutic intervention. The earliest study dates back to 2014, when researchers, using bioinformatics methods, identified Remodelin as a small molecule inhibitor of NAT10. The study demonstrated that Remodelin could inhibit NAT10 expression, showing therapeutic potential in conditions like HGPS and premature aging syndromes.^96^ Subsequent studies have revealed the targeted effects of Remodelin in other cancers, including liver cancer, leukemia, melanoma, and breast cancer.^35^, ^164^, ^207^, ^208^, ^209^, ^210^ To date, Remodelin has shown significant preclinical therapeutic efficacy in models of liver cancer, breast cancer, melanoma, and leukemia, primarily through NAT10 inhibition. However, due to current research and technical limitations, no other effective small‐molecule inhibitors of NAT10 have been identified aside from Remodelin.

Despite many researchers attempting alternative approaches, results have been mixed. For instance, in 2020, a team used ITC technology and protein thermal stability experiments to study the molecular dynamics of NAT10 and Remodelin interactions. Unfortunately, the results indicated no direct interaction between Remodelin and NAT10, nor did Remodelin affect intracellular RNA ac4C modification levels.^211^ Additionally, recent studies employed homology modeling to predict NAT10's protein structure and used virtual screening to explore potential NAT10 inhibitors from FDA‐approved drugs. The results identified two drugs, Fosaprepitant and Leucal, with excellent binding free energy to NAT10, significantly surpassing its natural substrate acetyl‐CoA. In comparison, the binding energy between Remodelin and NAT10 was notably lower than that of NAT10 and acetyl‐CoA. Unfortunately, for various reasons, researchers have not further explored these drugs’ interactions with NAT10, nor have they evaluated their effects on RNA ac4C modification levels or tested their therapeutic potential in vitro or in vivo models.^212^ Encouragingly, a recent promising study discovered lead compound #7586‐3507, which can target NAT10 Khib modification and inhibit cancer metastasis. In this research, the authors used multidisciplinary algorithms to screen and validate the ability of #7586‐3507 to inhibit the migration and invasion of esophageal squamous cell carcinoma by targeting NAT10. Unlike traditional inhibitors, this compound directly downregulated NAT10‐mediated ac4C levels in mRNA, marking a significant breakthrough. If further preclinical or clinical studies could be conducted to develop it into a clinically viable drug, the prospects would be promising.^34^

With the rapid advancement of artificial intelligence technologies, such as the AlphaFold2 protein structure prediction algorithm, new opportunities have emerged for drug development targeting NAT10 and its RNA ac4C modifications. AlphaFold2 can accurately predict the three‐dimensional structure of NAT10, revealing its interaction sites with RNA and other molecules, thus laying a foundation for drug design targeting this protein. Moreover, virtual screening technology allows rapid screening of large compound libraries through computer simulations, identifying small molecule drug candidates with high affinity for NAT10. Molecular dynamics techniques can further evaluate the binding stability and dynamic behavior of these small molecules with NAT10, predicting their in vivo activity and pharmacokinetic properties. In future research, integrating these technologies will significantly improve the efficiency of drug development targeting NAT10, reducing the time and costs associated with traditional drug discovery. Combining artificial intelligence with high‐performance computing for virtual screening and molecular dynamics simulations will accelerate the validation of candidate drugs and optimize their chemical structures to enhance drug selectivity and safety. Looking ahead, utilizing these cutting‐edge technologies for developing drugs targeting NAT10 and its RNA ac4C modification may offer more precise and personalized treatment options for cancer therapy, greatly improving clinical outcomes and patient prognoses.

## 8 CONCLUSIONS AND PERSPECTIVES

RNA ac4C modification is a highly conserved and functionally significant posttranscriptional modification, found across eukaryotes, archaea, and some bacteria. Primarily catalyzed by the enzyme NAT10, ac4C modification regulates RNA stability, structure, and translation efficiency, thereby influencing gene expression.^31^ Recent studies have demonstrated that ac4C modification plays a pivotal role in various biological processes, including the regulation of cell growth, differentiation, and stress responses.^35^ Moreover, ac4C modification is closely associated with the onset and progression of various diseases, particularly cancer and neurodegenerative disorders, presenting potential applications in diagnosis and therapy.^33^ As a result, ac4C modification is emerging as a key focus in the field of RNA modifications, holding significant implications for understanding RNA regulatory mechanisms and their roles in disease.

In this review, we comprehensively summarize the research trajectory, detection methods, and pivotal roles of RNA ac4C modifications in regulating RNA metabolism, including the modulation of mRNA stability, translation, rRNA biogenesis, and lncRNA stability. Additionally, we focus on the critical roles and mechanisms of NAT10‐mediated RNA ac4C modifications in regulating normal physiological processes, including stem cell fate, spermatogenesis, oogenesis, embryonic development, aging, DNA damage repair, cell cycle progression, chromosome decondensation, autophagy, and bone remodeling. Finally, we emphasize the core roles and molecular mechanisms of NAT10‐mediated RNA ac4C modifications in regulating tumor cell proliferation, cell cycle, metastasis and invasion, cell death, drug resistance, cellular metabolism, and tumor immunity. We highlight the effectiveness of NAT10‐mediated RNA ac4C modifications as disease diagnostic biomarkers and the potential of targeting NAT10 in various disease therapies, providing new perspectives on the potential of NAT10 as a diagnostic and therapeutic target in clinical applications. By summarizing the current research progress on NAT10 and ac4C modification, this review provides a systematic perspective on understanding the role of NAT10 in RNA modification and its involvement in related diseases, offering new insights and directions for future research in the fields of biology and medicine.

NAT10 and its mediated ac4C modifications hold significant potential and relevance as prognostic biomarkers for tumor diagnosis. NAT10 modifies RNA through ac4C, which affects gene expression regulation, and this modification is closely related to the onset and progression of various tumors. Studies have shown that specific ac4C modification patterns can serve as prognostic biomarkers for tumors, aiding in the identification of disease stages, predicting disease progression, and assessing patient outcomes.^213^ For instance, detecting ac4C modification levels in tumor tissues or bodily fluids can provide insights into tumor biological characteristics, guiding personalized treatment strategies.

The advantages of targeting NAT10 in tumor therapy include: first, as a crucial modification enzyme, NAT10‐targeted therapy could interfere with significant ac4C modifications in tumor cells, impacting cell proliferation, metastasis, and drug resistance reversal. Second, therapies targeting NAT10 might offer higher specificity, reducing damage to normal cells and thus improving treatment safety and efficacy. Additionally, targeting NAT10 could be combined with other therapeutic modalities, such as immunotherapy and chemotherapy, to enhance overall treatment efficacy.

However, targeting NAT10 in cancer therapy also faces certain limitations. First, the expression and role of NAT10 may vary among different tumor types, potentially affecting the universal applicability of the treatment. Second, research on NAT10‐targeted therapies is still in its early stages, and related drugs and treatment strategies require further optimization. Moreover, inhibition of NAT10 may lead to side effects, such as impacts on normal cell functions, necessitating careful safety assessments. Future research should focus on improving NAT10‐targeted therapy strategies to overcome these limitations. This includes developing more precise targeted drugs to enhance specificity and selectivity for NAT10. Additionally, understanding NAT10's role in different tumor types and subtypes will help optimize treatment strategies and predict outcomes. Furthermore, integrating emerging biomarkers and technologies, such as liquid biopsy and high‐throughput screening, could improve diagnostic accuracy and treatment personalization. These efforts could make NAT10‐targeted therapies a significant breakthrough in future cancer treatment.

There are several areas that still need advancement in the research of ac4C RNA modification technologies, particularly in improving detection methods, reducing costs, and increasing detection resolution and accuracy. For improving detection methods, more efficient and sensitive technologies need to be developed for the accurate identification and quantification of ac4C modifications. Current detection methods, while advanced, still have room for improvement in specificity and sensitivity. Future research could focus on optimizing existing techniques, such as mass spectrometry and ac4C‐RIP‐seq, and incorporating emerging single‐cell sequencing and spatial epitranscriptomics technologies to enhance detection capabilities. Additionally, developing high‐throughput and automated detection platforms could accelerate data acquisition and analysis. Enhancing detection resolution and accuracy is crucial for obtaining high‐quality data. Future research should aim to improve the spatial resolution and dynamic range of detection technologies to more precisely capture ac4C modifications in various cells and tissues. Implementing higher‐resolution microscopy techniques, advanced data analysis algorithms, and deep learning technologies will contribute to increased detection precision and reliability.

Currently, while research on NAT10 in tumors is extensive, the role of NAT10 in regulating the tumor immune microenvironment remains in its early stages, with many key events still unclear. Future research on NAT10's role in the tumor immune microenvironment should focus on several aspects. First, exploring the role of NAT10 in the tumor immune microenvironment is essential. As a significant modification enzyme, NAT10's unique function in regulating RNA ac4C modifications may influence immune cell function and tumor progression. Future studies should aim to elucidate how NAT10 affects immune cell activity and tumor immune evasion mechanisms through regulating immune‐related gene expression. This will aid in understanding NAT10's role in tumor immune evasion and provide a basis for developing new immunotherapy strategies. Second, more precise models and technologies should be developed to study the specific mechanisms of NAT10 in the tumor immune microenvironment. For example, single‐cell RNA sequencing, combined with analysis of NAT10 target genes and signaling pathways, can systematically explore NAT10's role in different tumor types. These studies will help reveal the specific mechanisms of NAT10 in tumor immune regulation and support the development of NAT10‐targeted immunotherapies. Additionally, future research should explore the potential of targeting NAT10 for therapy. Developing specific inhibitors or modulators and studying NAT10's regulatory role in the tumor immune microenvironment will help explore its potential as a novel immunotherapy target. Effective NAT10 inhibitors may improve the efficacy of immunotherapies, particularly for refractory tumors. In summary, future research should focus on elucidating NAT10's mechanisms in the tumor immune microenvironment, developing new research technologies and models, and exploring its potential as an immunotherapy target. These efforts will advance our understanding of tumor immune regulation and provide new strategies for developing more effective cancer treatments.

## AUTHOR CONTRIBUTIONS

Xiulin Jiang, Qiang Wang, and Yixiao Yuan wrote the main manuscript text. Qiang Wang, Yixiao Yuan, Qiang Zhou, YingDong Jia, and Jing Liu prepared the figures and analyzed the literature in depth and optimized the topic and structure. GuangJun Xiao developed the conception of the topic. Chunhong Li and Xiulin Jiang supervised, reviewed, and revised the written manuscript. All authors reviewed the manuscript.

## CONFLICT OF INTEREST STATEMENT

The authors declare no conflict of interest.

## ETHICS STATEMENT

Not applicable.

## Data Availability

Not applicable.
